# Molecular Targeting of Intracellular Bacteria by Homotypic Recognizing Nanovesicles for Infected Pneumonia Treatment

**DOI:** 10.34133/bmr.0172

**Published:** 2025-04-02

**Authors:** Xu Wang, Hao Zhou, Dan Li, Zhe Zhao, Ke Peng, Xiang Xu, Jia-Jia Wang, Yang Wang, Jun Wang, Jing-Jing Zhang, Shuang-Shuang Wan, Mai-Qing Shi, Jun Chen, Xian-Guang Ding, Fu-Hai Ji

**Affiliations:** ^1^ Department of Anesthesiology, The First Affiliated Hospital of Soochow University, Suzhou, Jiangsu, China.; ^2^ Department of General Surgery, The First Affiliated Hospital of Soochow University, Suzhou, Jiangsu , China.; ^3^ Nanjing University Medical School, Nanjing, Jiangsu 210008, China.; ^4^ Suzhou Institute of Nano-Tech and Nano-Bionics, CAS Key Laboratory of Nano-Bio Interface Chinese Academy of Sciences, Suzhou, Jiangsu, China.; ^5^ Department of Anesthesiology & Institute of Anesthesiology, The First Affiliated Hospital of Soochow University, Suzhou, Jiangsu, China.; ^6^ Department of Neurosurgery & Brain and Nerve Research Laboratory, The First Affiliated Hospital of Soochow University, Suzhou, Jiangsu, China.; ^7^ Department of Pulmonary and Critical Care Medicine, The First Affiliated Hospital of Soochow University, Suzhou, Jiangsu, China.; ^8^ Department of Intensive Care Medicine, The First Affiliated Hospital of Soochow University, Suzhou, Jiangsu, China.; ^9^ State Key Laboratory of Organic Electronics and Information Displays & Jiangsu Key Laboratory for Biosensors, Nanjing University of Posts and Telecommunications, Nanjing, Jiangsu 210023, China.

## Abstract

Although extensive antibiotic regimens have been implemented to address pathogen-infected pneumonia, existing strategies are constrained in their efficacy against intracellular bacteria, a prominent contributor to antibiotic resistance. In addition, the concurrent occurrence of a cytokine storm during antibiotic therapy presents a formidable obstacle in the management of pneumonia caused by pathogens. In the present study, an infection-targeting system that leverages M2-macrophage-derived vesicles [exosomes (Exos)] as vehicles to convey antibiotics (antibiotics@Exos) was developed for effective pneumonia management. The proposed system can enable antibiotics to be specifically delivered to infected macrophages in pneumonia through homotypic recognition and was found to exhibit an exceptional intracellular bactericidal effect. Moreover, M2-type vesicles exhibit a high degree of efficiency in reprogramming inflammatory macrophages toward an anti-inflammatory phenotype. As a result, the administration of antibiotics@Exos was found to substantical decrease the level of the infiltrated inflammatory cells and alleviate the inflammatory factor storm in the lungs of acute lung injury mice. This intervention resulted in the alleviation of reactive-oxygen-species-induced damage, reduction of pulmonary edema, and successful pneumonia treatment. This bioactive vesicle delivery system effectively compensates for the limitations of traditional antibiotic therapy regimens with pluralism effects, paving a new strategy for serious infectious diseases, especially acute pneumonia treatment.

## Introduction

Pneumonia represents a widespread and challenging pulmonary ailment. Globally, nearly 600 million cases of pneumonia and other lower respiratory tract infections occur annually, resulting in approximately 2.5 million fatalities each year [1]. The severity of pneumonia is mainly determined by the following 3 factors: (a) pathogens nestling in phagocytic cells and biofilms, which can reduce exposure to the antibiotic leading to antimicrobial resistance and escape from host defenses [2]; (b) insufficient antibiotic concentration at the inflamed area of the lung [3,4]; and (c) inevitable host inflammatory response triggered by microorganisms that can cause severe immune dysregulation and injury of alveolar endothelium [5]. Despite the development of extensive antibiotic regimens, the prognosis for individuals with severe pneumonia remains suboptimal [6]. Hence, there is an urgent need for new strategies to overcome the existence of intracellular bacteria and avoid cytokine storms during pneumonia treatment.

Recent evidence has indicated that macrophages can serve as pathogen reservoirs and impede the penetration of antibiotics for bacteria treatment after engulfing invading pathogens [7,8], resulting in widespread antibiotic resistance. Pathogens can effectively evade macrophage destruction through mechanisms such as the expression of virulence factors, adaptation to the phagosomal environment, or escaping from the phagosome and persisting in the cytosol [9]. These lead to bacteria remaining within phagocytes for extended periods, leading to pathogen propagation and accumulation of toxicity within the cell. Hence, one of the typical strategies of antimicrobial treatment is to increase the concentration of antibiotics in phagocytes to achieve the desired purpose of intracellular sterilization. Further, the development of pneumonia can be attributed to the dysregulation of the proinflammatory (M1-type) and anti-inflammatory (M2-type) environments induced by pulmonary macrophages, serving as another significant contributing factor [10,11]. Bacteria’s lipopolysaccharide (LPS) has the capacity to induce the differentiation of macrophages into M1-type macrophages by interacting with Toll-like receptors [12]. It releases cytokines that can inhibit the proliferation of surrounding cells and damage adjacent tissue, such as tumor necrosis factor-α (TNF-α) and reactive oxygen species (ROS) [13]. Nonetheless, M2-types can produce interleukin-10 (IL-10) and transforming growth factor-β to trigger tissue repair and remodeling via the promotion of anti-inflammatory and immunosuppressive responses [14]. An avenue of promising therapeutic strategies centered around macrophage polarization has emerged in the context of fibrosis, hepatitis, colitis, and tumor progression [15]. Therefore, repolarizing macrophages from proinflammatory M1-types to anti-inflammatory M2-types holds the therapeutic promise to reduce the secretion of harmful cytokines and may play a crucial role in pneumonia progression and recovery.

Exosomes (Exos) are small, 50- to 200-nm membraned vesicles secreted by various cell types in the extracellular milieu, for example, immune cells, nerve cells, and tumor cells [16]. Recent studies have suggested that Exos encompass the advantages of both synthetic carriers and cell-mediated carriers [17]. Compared with liposomes and nanocarriers, Exos offer several advantages, including low immunogenicity, noncytotoxicity, stability, and high biocompatibility [18,19]. Therefore, Exos possess superior drug delivery potential while also inheriting the properties of parental cells [20,21]. Exos share similar phospholipid bilayer structures and specific membrane proteins and lipids with their parental cells on their surface, which can promote their fusion with parental cells. This feature has 3 significant benefits in drug delivery [21–23]: (a) It promotes the effective targeting of disease tissues, enhancing the precision of therapeutic interventions; (b) Exos possess the capability to carry ample amounts of antibiotics, ensuring a sufficient payload for combating infections; (c) they may contain key regulators responsible for orchestrating cell differentiation and polarization processes, potentially influencing therapeutic outcomes. In particular, M2-macrophage-derived Exos (M2-Exos) play an anti-inflammatory role in inflammatory response by orchestrating the reprogramming of monocytes [10,24].

**Fig. 1.**
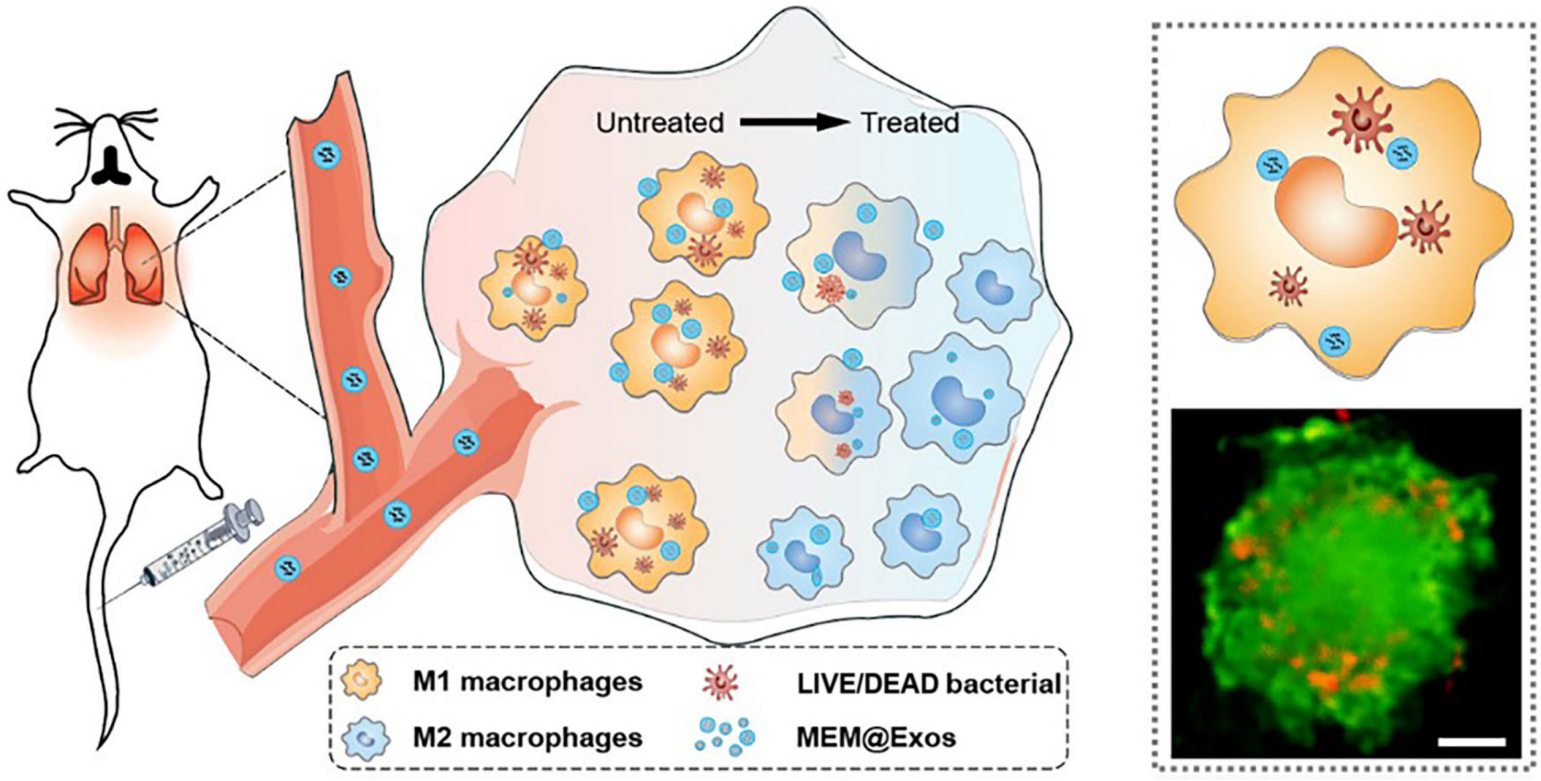
M2-macrophage-derived vesicles (Exos) serve as vectors for delivering antibiotics (antibiotics@Exos) to infected macrophages in pneumonia and exhibit exceptional intracellular bactericidal efficacy. The inserts show the intracellular sterilization of antibiotics@Exos. The red fluorescence in the cell image (green stained) indicates intracellular deceased bacteria. Scale bar, 5 μm.

Inspired by recent studies on Exos’ efficacy in treating inflammation [25], an infection-targeting system to improve severe pneumonia treatment was developed in the present study. Antibiotic meropenem (MEM) was loaded with M2-Exos to form MEM@Exos. In contrast to conventional antibiotic regimens used for loading antibiotics in pneumonia therapy, this technology represents a substantial advancement that markedly enhances therapeutic outcomes against intracellular bacteria and alleviates inflammation-induced damage to lung tissues (Fig. 1). Consequently, it significantly augments the efficacy of pneumonia treatment.

## Materials and Methods

### Preparation of Exos

To prepare M1-types, around 2.0 × 10^6^ of RAW264.7 cells were seeded in 100-mm culture dish and stimulated with interferon-γ (100 ng/ml) for 24 h. The liquid supernatant was discarded after centrifugation at 500*g* (10 min). M1-type macrophages were suspended in phosphate-buffered saline (PBS) and subsequently stored at −80 °C for long-term preservation.

To prepare M2-types, around 2.0 × 10^6^ of RAW264.7 cells were seeded in 100-mm culture dish and stimulated with IL-4 (20 ng/ml) for 24 h. The medium was centrifuged at 1,000*g* for 10 min to remove cellular debris. The supernatant medium was carefully transferred into new tubes and centrifuged at 5,000*g* for 20 min to remove the apoptotic corpuscles. Subsequently, a Millipore membrane filter (SLGP033RB, Millipore) with 0.22-μm pore size was used to remove large extracellular vesicles. After that, M2-Exo pellets were resuspended in PBS and ultracentrifuged again at 120,000*g* for 2 h to wash away the contaminating protein. At this point, M2-Exos were obtained. M2-Exos were resuspended into PBS and placed at −80 °C for long-term storage. All centrifugation steps were conducted at 4 °C.

### Construction and characterization of MEM@Exos

A total of 20 μg of purified M2-Exos were combined with 50 μg of MEM, and the mixture was subjected to electroporation using a 4-mm electroporation cuvette (X-Porator H1) at 1,000 kV for 5 ms. To remove any unloaded MEM, the medium was washed twice with PBS. The resulting MEM@Exos were incubated at 37 °C for 30 min. Next, precipitant (System Biosciences, ExoQuick-TC) was added at a 5:1 ratio, and the mixture was allowed to stand overnight at 4 °C to facilitate the recovery of the Exo membrane. Finally, the purified MEM@Exos were resuspended in an equal volume of PBS, making them ready for use. To create the control liposomes for delivering MEM, the lipid composition matched that of MEM@Lip (lipidosome-MEM), consisting of 70% 1,2-dioleoyl-*sn*-glycero-3-phosphocholine (DOPC) and 30% cholesterol. Specifically, 28 μmol of DOPC and 12 μmol of cholesterol were dissolved in 1 ml of ethanol, thoroughly mixed, and dried under a stream of nitrogen gas. The MEM loading process used the same electroporation strategy as used for MEM@Exos, ensuring consistency in preparation. The loading efficiency of MEM into Exos was quantified using high-performance liquid chromatography (HPLC). The amount of MEM encapsulated in the exosomal preparation was compared to the initial MEM concentration to calculate the loading efficiency. The release profile of MEM from MEM@Exos under acidic conditions was assessed using in vitro release assays in a pH 5.5 buffer. The amount of MEM released was quantified using HPLC over several time points to evaluate drug stability and release kinetics in a simulated acidic environment.

The morphology and function of the prepared MEM@Exos were tested. The size distribution and zeta potential were determined using dynamic light scattering (DLS) (Zetasizer 3000HS, Malvern, UK). The morphology and 3-dimensional structure of particles were characterized using transmission electron microscopy (TEM; HT7700, Hitachi Ltd., Tokyo, Japan).

The exosomal markers CD81 and CD63 were detected using Western blot (WB) analysis. Cells were lysed in radioimmunoprecipitation assay buffer (Beyotime Biotech, China) and quantified with a bicinchoninic acid protein assay kit (KeyGEN BioTECH, China). The separation of CD81 and CD63 proteins on polyvinylidene difluoride (PVDF) membranes was accomplished using SDS–polyacrylamide-gel-electrophoresis. Subsequently, the PVDF membranes were blocked with fat-free dried milk in PBS-Tween 20 at 37 °C for 45 min. Then, the membranes were incubated with CD81 and CD63 antibodies at 4 °C overnight. After washing 5 times intris-phosphate-buffered saline (TBS-T), membranes were incubated with the horseradish-peroxidase-conjugated secondary antibody (ProteinSimple, Bio-Techne, USA) at 4 °C for 30 min. A gel imaging system (Azure C300, Azure Biosystems Inc.) was used to visualize immunoreacted proteins.

### Animals, cell, and *Escherichia coli* culture

All live animal experiments were conducted under the guidance of the Academic Ethics and Ethics Committee of Nanjing University of Posts and Telecommunications. BALB/c mice (female, 18 to 20 g) were purchased from the experimental animal center of Yangzhou University.

Human umbilical vein endothelial cells (HUVECs) and the macrophage cells RAW264.7 were obtained from Cell Bank for Nanjing University of Posts and Telecommunications. All cell cultures were maintained in Dulbecco’s modified Eagle’s medium (Invitrogen, USA) supplemented with dextrose (4.53 g/l), 10% fetal bovine serum, and 1% penicillin and streptomycin, within a controlled environment of 5% CO_2_ at 37 °C.

*Escherichia coli* (APEC-O78 strain) was obtained from the National Center for Veterinary Culture Collection (Beijing, China) and stored at −80 °C in the refrigerator. *E. coli* was cultured in Luria–Bertani broth (Qingdao Hope Bio Technology Co. Ltd., Qingdao, China) on a shaker at 37 °C for about 12 h at 200 rpm. Then, the bacteria culture medium was centrifuged, and bacterial pellets were resuspended in PBS to produce the inoculums.

### Hemolysis assay to evaluate red blood cells membrane stabilization

Human red blood cells (RBCs) hemolytic assay was conducted to test the hemolytic potential of MEM@Exos using fresh isolated RBCs. A total of 5 ml of whole blood was centrifuged at 4,000*g* rpm for 10 min to separate the packed erythrocytes from the plasma. A total of 200 μl of isolated erythrocytes was diluted with 0.9 ml of PBS (pH 7.4) to prepare the suspension of erythrocytes. The RBC suspensions were then individually mixed with about 100 μl of different concentrations of MEM@Exos. Subsequently, 2 ml of ultrapure water or PBS (pH 7.4) was added to 0.2 ml of diluted RBC suspensions as the positive and negative control groups, respectively. All the samples were incubated at 37 °C for 1 h before being centrifuged at 4,000*g* rpm for 10 min. A total of 100 μl of the supernatant was taken and transferred to a 96-well plate, and the absorbance of hemoglobin was measured using a microplate reader (*λ* = 540 nm). The percentage of hemolysis was calculated using the following equation: hemolysis (%) = [(*A*_sample_ − *A*_negative_) / (*A*_positive_ − *A*_negative_)] × 100%.

### Cytotoxicity assay

To detect the cytotoxicity of MEM@Exos, Cell Counting Kit-8 (CCK-8) assay was used. HUVECs were seeded into a 96-well plate at 4 × 10^3^ per well and incubated with various doses of MEM@Exos. After 0, 24, 48, 72, and 96 h, the medium was removed, and a fresh medium containing CCK-8 (Biyuntian Company, Shanghai, China) was introduced. The optical density value of the incubation at 450 nm was measured after 2 h. This assay was repeated 3 times, and the data were calculated using the average results. Subsequently, the treated cells were stained with calcein acetylmethyl (AM) (2 μmol/ml; *λ*_excitation_/*λ*_emission_ = 490 nm/515 nm) and propidium iodide (PI) (4.5 μmol/ml; *λ*_excitation_/*λ*_emission_ = 530 nm/580 nm). When observed under a fluorescence microscope, red fluorescence indicated the presence of dead cells, while green fluorescence signified the presence of living cells.

### Cellular uptake of MEM@Exos

The fluorescent 1,1′-dioctadecyl-3,3,3′,3′-tetramethylindodicarbocyanine perchlorate (DiD) (Invitrogen, Carlsbad, CA) was used to label the membranes of MEM@Exos and MEM@Lip. After labeling, PBS was used to wash the samples twice, followed by centrifugation to remove any residual free dye. M1-types were incubated with DiD-labeled MEM@Exos or MEM@Lip at 37 °C in 5% CO_2_ for 24 h. The M1-types were fixed with 4% (v/v) paraformaldehyde for 20 min, washed 3 times with PBS, and labeled with the fluorescent dye, 3,3′-dioctadecyloxacarbocyanine perchlorate (DiO) (Beyotime, Jiangsu, People’s Republic of China). After staining the nucleus with 10% Hoechst for 15 min, the residual dye was removed. Finally, fluorescence microscopy was used to observe the intracellular distributions of MEM@Exos and MEM@Lip.

### Imaging in vivo

The acute lung injury (ALI) mouse model was established according to previous studies by instilling intranasally with 40 μl of inoculum of *E. coli* (containing approximately 4 × 10^9^ colony-forming units) as prepared in advance. After 5 h, DiD-labeled MEM@Exos and MEM@Lip were administered by means of intravenous injection, respectively. As a control, DiD-MEM@Exos was also injected intravenously into healthy mice. After 4 h, the mice were euthanized. The lung of the mice was imaged using an imaging system (IVIS Lumina Series III, PerkinElmer Inc., USA) to analyze the total flux intensities (in photons per second). To assess material retention, mouse organs were harvested 24 h later.

### The bactericidal ability of MEM@Exos against live *E. coli* in antibiotics in vitro

RAW264.7 cells were plated at a density of 4 × 10^5^ cells per well and infected with *E. coli* at a ratio of 10 to 20 bacteria per macrophage. After 24 h, the medium was cleaned with PBS to remove free *E. coli*. The infected macrophages were cocultured with MEM@Exos (20 μg/ml equivalent MEM) for 0, 3, 6, 9, 12, and 15 h and exposed to diverse concentrations of MEM@Exos with 0, 10, 50, and 100 μg/ml. Then, washing with PBS was performed twice to remove the supernatant. The macrophages were stained with wheat germ agglutinin-Alexa Fluor 633 (10 μg/ml) for 15 min and fixed with 4% paraformaldehyde for 30 min. Washing with sterile PBS was performed twice before perforation with 0.1% (v/v) Triton X-100 for 5 min. Washing with sterile PBS was performed 3 times before incubation with LIVE/DEAD BacLight bacterial viability diluent (KeyGEN BioTECH, China) at room temperature for 20 min. The cells were rinsed 3 times with PBS, and *E. coli* was observed using laser cofocus microscopy (Leica TCS SP8, Solms, Germany).

### Flow cytometry

After *E. coli*-infected M1-type macrophages were cocultured with PBS, MEM, M2-Exos, and MEM@Exos for 24 h, the cells were collected before incubation with phycoerythrin (PE)–anti-CD11b, PE–anti-CD80, and PE–anti-CD206 antibodies (BioLegend, San Diego, CA) at 37 °C. The cells were rinsed with precooling PBS twice and then analyzed using flow cytometry. Flow cytometry data were acquired using a BD LSR II (Franklin Lakes, NJ, USA), and data analysis was performed with FlowJo X.

### Immunofluorescence staining of lung tissues

Ten-micrometer frozen sections of lung tissues from ALI mice were obtained using a Leica CM1860 cryostat. These sections were allowed to air-dry for a duration of 20 min, followed by a 20-min postfixation step in 100% methanol at a temperature of −20 °C. After washing in TBS-T, the sections were permeabilized using 0.2% Triton X-100 in TBS-T for 20 min at room temperature. Washed sections were incubated for 30 min in blocking solution (3% bovine serum albumin and 10% normal goat serum in TBS-T). The sections were incubated with rabbit anti-mouse CD206 at a 1:500 dilution and incubated overnight at 4 °C. The cells were incubated with the fluorescence-labeled anti-rabbit secondary antibody (1:1,000) for 1 h in a dark chamber at room temperature. Sections were thoroughly washed 5 times for 15 min in TBS-T before mounting with antifade mountant with 4′,6-diamidino-2-phenylindole (DAPI) (Thermo Fisher Scientific). Finally, images were collected under a fluorescence microscope (Nikon, Tokyo, Japan). The collected images were quantified using Image-Pro Plus 6.0 (Media Cybernetics, Silver Spring, MD, USA). The blue-stained part was the nucleus, and the red fluorescence indicated the number of CD206^+^ cells.

### Enzyme-linked immunosorbent assay to detect IL-10 and TNF-α

After successful induction of the ALI model with *E. coli*, the mice were divided into 4 groups (8 per group) treated with PBS, MEM, M2-Exos, and MEM@Exos (20 μg/ml) by intravenous administration. After 24 h, the mice were euthanized to obtain the whole blood and the lungs. Serum was isolated from whole blood, and inflammatory factors were detected (IL-10 and TNF-α) according to the standard protocol of the enzyme-linked immunosorbent assay (ELISA) kit (Tianjin Anoric Biotechnology, Tianjin, China).

### Lung wet-to-dry weight ratio

To evaluate the extent of pulmonary edema, fresh lung tissues were weighed in vivo to determine their wet weight. Subsequently, the lungs were dried overnight at a temperature of 60 °C to obtain their dry weight. The ratio of lung wet weight to dry weight was calculated to assess the degree of pulmonary edema.

### Hematoxylin and eosin staining of lung tissues and leukocyte marker CD45 in lung tissues

The inflammatory infiltrate was characterized by the immunohistochemistry of staining for the leukocyte common antigen CD45. Polyclonal antibody specific for CD45 (GB11522, Servicebio) was used as the primary antibody, and secondary labeling was performed using 3,3′-diaminobenzidine​ (DAB) chromogenic kit (Dako). Images were acquired using light microscopy (Nikon DS-U3) to evaluate leukocyte infiltration.

### ROS assay

Superoxide production in the ALI was evaluated with the dihydroethidium (DHE) for detecting ROS. Fresh lungs were kept at −80 °C and cut into frozen sections, which were incubated with DHE at 37 °C for 40 min and fixed with paraformaldehyde for 10 min. DAPI staining solution was used to stain the nucleus for 10 min. Finally, the images were examined using a fluorescence microscope. The blue staining represented the nucleus, while the red fluorescence indicated the content of ROS. A consistent exposure time of 30 ms was used for image acquisition across all sections.

### The major organs slice with H&E staining

Major organs were fixed in 4% paraformaldehyde and sliced into paraffin sections for hematoxylin and eosin (H&E) staining to evaluate the toxicity of organs, including the heart, liver, spleen, and kidney.

### Statistical analysis

All data were presented as means ± SD. Statistical significance was calculated by means of one-way analysis of variance (ANOVA) using Tukey’s test. The SPSS 26.0 program was used for statistical analysis. *P < 0.05, **P < 0.01, ***P < 0.001, and ****P < 0.0001 were considered statistically significant.

## Results

### Preparation and characterization of MEM@Exos

**Fig. 2.**
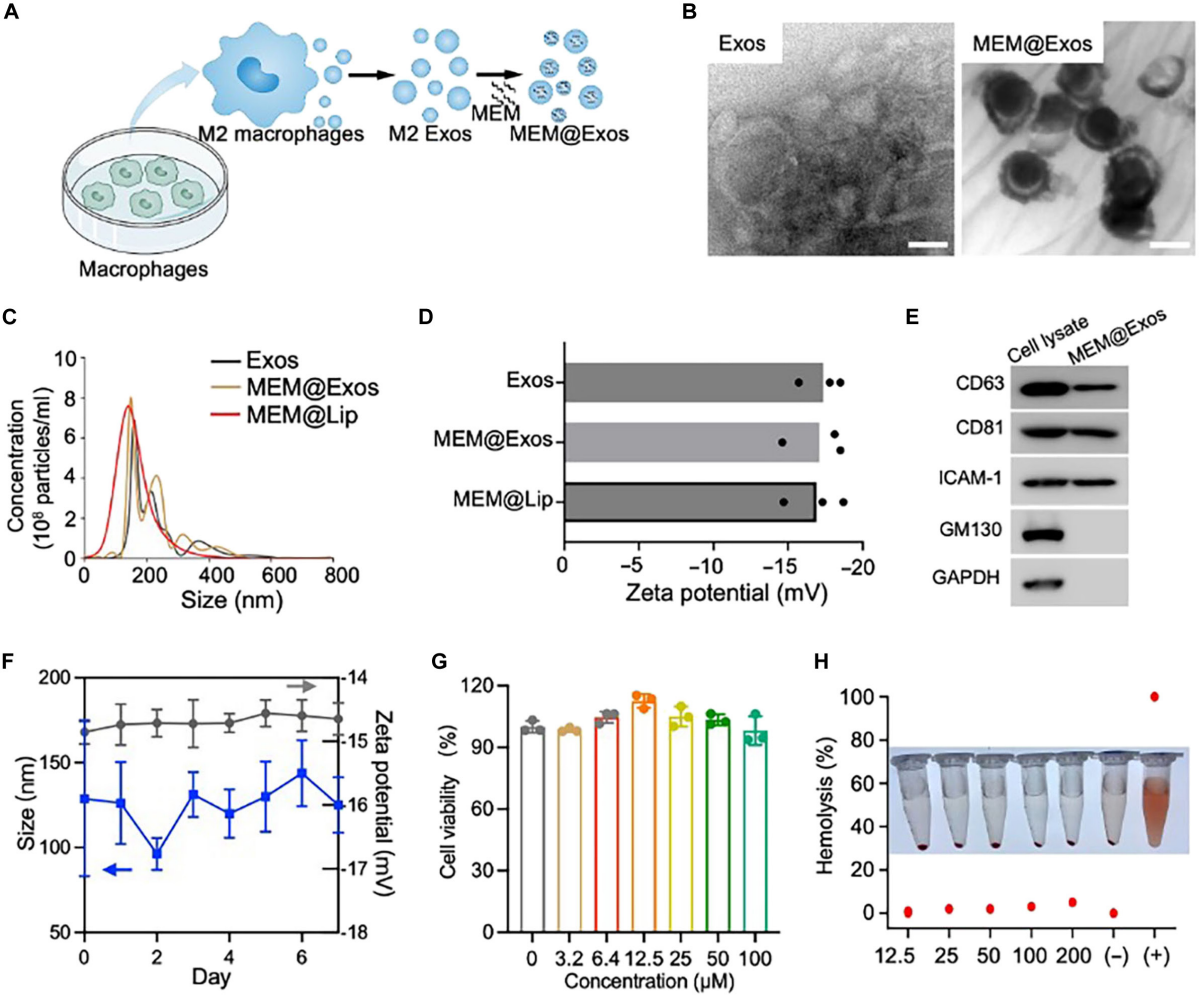
(A) An electroporation strategy was used to load antibiotic MEM into M2-Exos to form MEM@Exos. (B) TEM image of M2-Exos and MEM@Exos. Scale bars, 100 nm. (C) The size of M2-Exos, MEM@Exos, and MEM@Lip measured using nitrilotriacetic acid. (D) Zeta potential of M2-Exos, MEM@Exos, and MEM@Lip as compared by DLS. (E) The expression of exosomal specific markers (CD63, CD81, and ICAM-1) in the WB analyses. GAPDH, glyceraldehyde-3-phosphate dehydrogenase. (F) The stability of M2-Exos and MEM@Exos. After 1 week of dispersion, the size and zeta potentials were measured using DLS. (G) Cytotoxicity assay. CCK-8 assay was used to detect cytotoxicity in HUVECs of different dosages of MEM@Exos. (H) Hemolysis assay. A hemolytic assay was conducted to test the hemolytic potential of MEM@Exos using human RBCs. Ultrapure water or PBS (pH 7.4) was added to diluted RBC suspensions as the positive and negative control groups, respectively.

To obtain M2-Exos, M2-type macrophages were first obtained by stimulating RAW264.7 macrophages with IL-4. The typical M2 biomarker CD206 was found to be significantly increased after IL-4 stimulation for 12 h (*P* < 0.01) (Fig. S1). Subsequently, M2-macrophage-derived Exos were collected by ultracentrifuging the supernatant. An electroporation strategy was then used to load antibiotic MEM into M2-Exos to form MEM@Exos (Fig. 2A). TEM images of the obtained particles suggested that MEM@Exos and M2-Exos shared similar morphologies (Fig. 2B), both of which showed spherical structures with sizes ranging from 50 to 300 nm (Fig. 2C). Compared with M2-Exos, MEM@Exos maintained similar zeta potentials of around −16 mV to pure Exos (Fig. 2D), suggesting that the loading of MEM molecules did not exert any significant influence on the zeta potential of the Exo surface. The corresponding loading efficiency of MEM was calculated to be 25.7% under electroporation treatment, which was higher than incubation and comparable to the extrusion method. However, high-pressure processes may cause damage to Exos and loss of vesicle content. Therefore, electroporation was chosen for loading MEMs. The WB results show typical expression of exosomal markers CD63 and CD81 in MEM@Exos (Fig. 2E).

The obtained MEM@Exos demonstrated exceptional stability, as revealed by DLS and zeta potential measurements, which showed no significant differences even after 1 week of dispersion (Fig. 2F). The release profile of MEM@Exos demonstrated that acidic conditions, such as those found in endosomes, could enhance the release of MEM (Fig. S2). The cytotoxicity of MEM@Exos was further evaluated with HUVECs as model normal cells. The corresponding CCK-8 assay and AM/PI staining indicated that there was no apparent difference in HUVEC activity between the MEM@Exo group and the control group at a MEM@Exo dosage of 100 μM/ml (Fig. 2G and Fig. S3), indicating that MEM@Exos had no obvious cytotoxicity. Further, it was found that no significant hemolysis occurred until the dose of MEM@Exos was increased to 200 μM/ml, while large amounts of hemoglobin were observed in the supernatant in positive controls (Fig. 2H), suggesting that the MEM@Exos did not increase the risk of homeostasis after intravenous injection, potentiating their systemic administration for disease treatment.

### Inflammation targeting of MEM@Exos

**Fig. 3.**
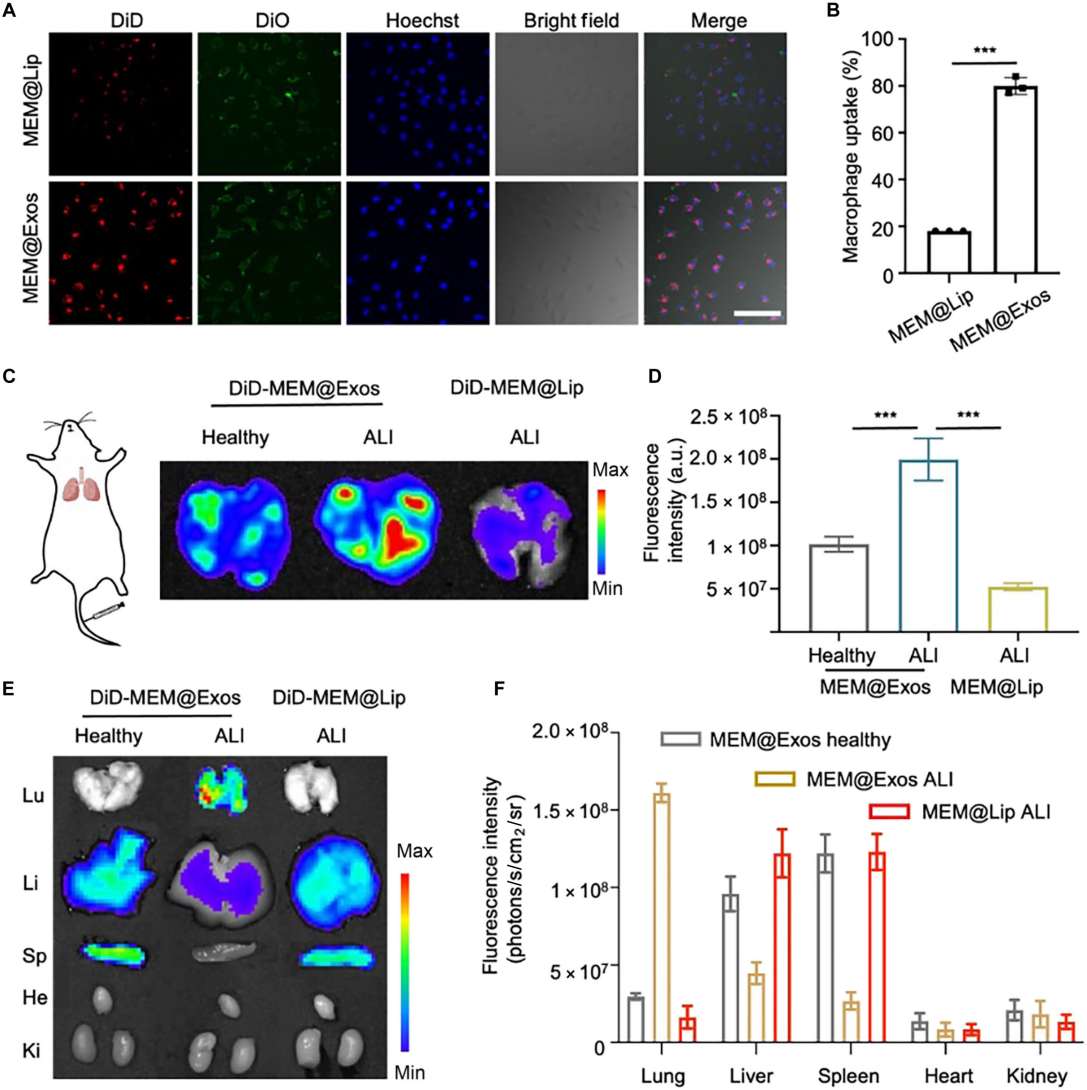
(A) MEM@Exos was phagocytosed by inflammatory M1-types. The intracellular distributions of MEM@Exos and MEM@Lip by fluorescence microscopy. Labeling: MEM@Lip and MEM@Exos with DiD (red), M1-types with DiO (green) and the nucleus with Hoechst (blue). Scale bar, 100 μm. (B) The rate of macrophage uptake for MEM@Exos and MEM@Lip (***P < 0.001). (C) Inflammation targeting of MEM@Exos in vivo. DiD-MEM@Exos and DiD-MEM@Lip were injected into healthy or *E. coli*-induced ALI mice through the tail vein, respectively. As a control, DiD-MEM@Exos were also injected intravenously into healthy mice. After 4 h, lung tissues were harvested for fluorescent analysis by imaging in vivo. DiD-MEM@Exos and DiD-MEM@Lip in the lungs with healthy and ALI mice. (D) Fluorescence quantitative analysis in the lungs of different groups. ***P < 0.001. a.u., arbitrary units. (E) Fluorescent images of major organs of mice treated with DiD-MEM@Exos and DiD-MEM@Lip after 24 h, respectively. (F) The fluorescence intensity in major organs (lungs, liver, spleen, heart, and kidney) of ALI and healthy mice in different groups after 24 h.

To determine the inflammatory chemotaxis capability of M2-Exos for infection recognition, the initial investigation focused on evaluating their ability to target inflammatory macrophages. As a control, liposomes with a comparable size to Exos were also constructed to load MEM (MEM@Lip). Both MEM@Exos and MEM@Lip were labeled with DiD dye (red fluorescent) and incubated with M1-type macrophages (DiO-labeled, green fluorescent) to evaluate their internalization by inflammatory macrophages. As shown in Fig. 3A, compared with the MEM@Lip group, MEM@Exos were significantly phagocytized by inflammatory M1 macrophages, with a phagocytic ratio up to 80% (Fig. 3B). The merged imaging well clearly depicted the presence of M2-Exos within the cytoplasm of macrophages. We further conducted intracellular trafficking studies using fluorescence-labeled Exos to track their internalization and antibiotic release within macrophages. These studies reveal that Exos are efficiently internalized via endocytic pathways and the antibiotics are gradually released in the phagosome (Fig. S4). This intracellular internalization study underscores the capacity of M2-Exos to significantly augment the phagocytosis of endothelial cells, thus enhancing their drug delivery capabilities for targeted inflammatory responses.

To further investigate the inflammatory chemotaxis of M2-Exos in vivo, DiD-MEM@Exos and DiD-MEM@Lip were injected into healthy or *E. coli*-induced ALI mice through the tail vein, respectively. After 4 h, the mice were euthanized, and the lung tissues were harvested for fluorescent analysis. Compared with healthy and ALI mice treated with DiD-MEM@Lip, the DiD-MEM@Exo treatment group exhibited the strongest fluorescence signal in ALI mice (Fig. 3C). Such findings were further confirmed by quantitative analysis (Fig. 3D), indicating that MEM@Exos had exceptional inflammation–tropism capability in vivo. After 24 h, the fluorescence signal demonstrated slight attenuation in the lungs; however, the intensity quantification in the DiD-MEM@Exo group was still much higher than the other 2 groups (Fig. 3E). Meanwhile, in both the healthy and DiD-MEM@Lip groups, there were no substantial variations in the distribution of fluorescence signals, primarily concentrated in the liver and spleen for drug clearance. Minimal signal was detected in the lungs, heart, and kidney (Fig. 3F). We also conducted MEM plasma concentration analysis following intravenous administration of MEM@Exos. The result demonstrated that drug signal maintained until 12 h (Fig. S5).

Owing to the expression of chemokine receptors such as chemokine ligand 1 (CCL1), CCL18, and intercellular adhesion molecule-1 (ICAM-1) on M2-Exos, they can exhibit high recruitment of infection sites, thereby effectively solving the problem of poor targeting of existing Exos [26–28]. The present results reveal that M2-Exos can target the inflammation site and be efficiently internalized by inflammatory macrophages, indicating the exceptional biocompatibility of MEM@Exos. At the same time, they exhibited the trait of persisting at the pneumonia site for an extended duration without undergoing clearance.

### Intracellular bactericidal effect of MEM@Exos

Intracellular infection treatment has emerged as the biggest challenge for microbial therapy in clinics. While most bacteria engulfed by phagocytic cells are degraded by enzymes by form “phagocytic lysosome” with lysosomes [29], a portion of intracellular bacteria can evade phagocytosis and persist within phagosomes for an extended duration, with the potential to invade other nonphagocytic cells [30,31]. Thus, a thorough treatment of infection would require the effective killing of intracellular bacteria.

**Fig. 4.**
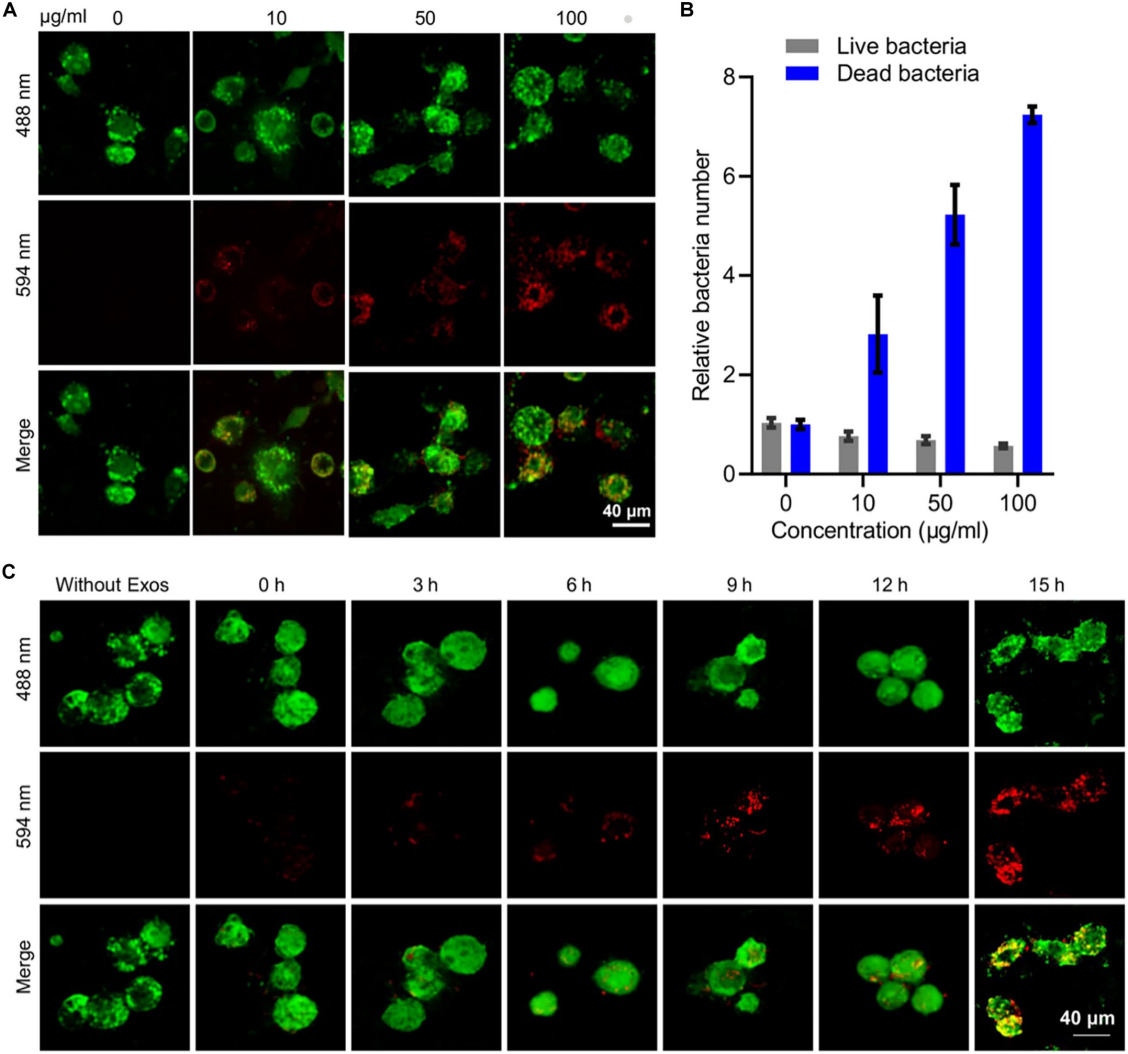
(A) The infected macrophages with *E. coli* were exposed to diverse concentrations of MEM@Exos (0, 10, 50, and 100 μg/ml). The LIVE/DEAD BacLight bacterial viability assay was used to distinguish intracellular dead bacteria (labeled in red) and live bacteria (labeled in green). The membrane of macrophages was stained with wheat germ agglutinin-Alexa Fluor 633 (green). Scale bar, 40 μm. (B) The relative number of live and dead *E. coli* in RAW264.7 cells after being treated by diverse concentrations of MEM@Exos (0, 10, 50, and 100 μg/ml). (C) The infected macrophages by *E. coli* were cocultured with MEM@Exos (20 μg/ml equivalent MEM) for 0, 3, 6, 9, 12, and 15 h and then stained with a LIVE/DEAD BacLight kit. Scale bar, 40 μm.

Motivated by the superiority of M2-Exo-targeting macrophages, further verification was performed to determine whether MEM@Exos had the effect of intracellular sterilization. Confocal microscopy analyses were performed on infected macrophages treated with MEM@Exos. First, macrophages and *E. coli* were cocultured. The LIVE/DEAD BacLight bacterial viability assay was used to distinguish intracellular dead bacteria (labeled in red) and live bacteria (labeled in green) (Fig. S6A). Different concentrations of MEM@Exos (10, 50, and 100 μg/ml) were coincubated with *E. coli*-infected macrophages compared with free MEM. The results indicate that with the increase in MEM@Exo concentration, the intracellular bactericidal effect was most significant upon reaching 100 μg/ml (Fig. 4A and B). Further, the effect of time gradient on sterilization was also explored (Fig. 4C). In the free MEM group, almost no intracellular bacterial death was observed within 9 h after treatment (Fig. S6B and C). Conversely, in the MEM@Exo group, intracellular dead bacteria were found after 3 h of coincubation, and the number of killed bacteria increased along with the extension time. At the 15-h mark, the number of deceased bacteria exceeded that of live ones by a factor of 8 (Fig. S7). We further performed additional experiments to validate the efficacy of MEM@Exos against *Staphylococcus aureus* (MRSA). In an in vitro model of intracellular infection, macrophages infected with *S. aureus* were treated with MEM, MEM@Lip, or MEM@Exos. Using the LIVE/DEAD BacLight assay, we observed that MEM@Exos exhibited significantly higher intracellular bactericidal activity compared to free MEM or MEM@Lip (Fig. S8). Specifically, dead bacteria (red-stained) were predominantly observed in MEM@Exo-treated cells, whereas live bacteria (green-stained) persisted in other groups. These results confirm that MEM@Exos can effectively target and eliminate intracellular *S. aureus*.

These findings demonstrate that elevating the concentration and extending the treatment duration of MEM@Exos can effectively eliminate intracellular bacteria. Further, the utilization of M2-Exos offers a novel approach to addressing the challenge of antibiotic resistance.

### MEM@Exos reprogram inflammatory macrophages toward M2-types

The cell walls of Gram-negative bacteria, led by *E. coli*, all contain LPS components. These LPS components have the capacity to trigger the inappropriate activation of the immune system, prompting macrophages to polarize into M1-types and consequently leading to an exaggerated inflammatory response [32,33]. Induced phenotypic transformation of macrophages from M1 to M2 can effectively prevent lung injury [10,34]. As previously reported, macrophages can be polarized by means of exogenous therapeutic methods to complete the conversion between M1 and M2 phenotypes and ultimately play a therapeutic role [35,36]. Hence, in vitro and in vivo experiments were conducted to verify whether M2-Exos could reprogram the transformation of macrophage phenotypes.

**Fig. 5.**
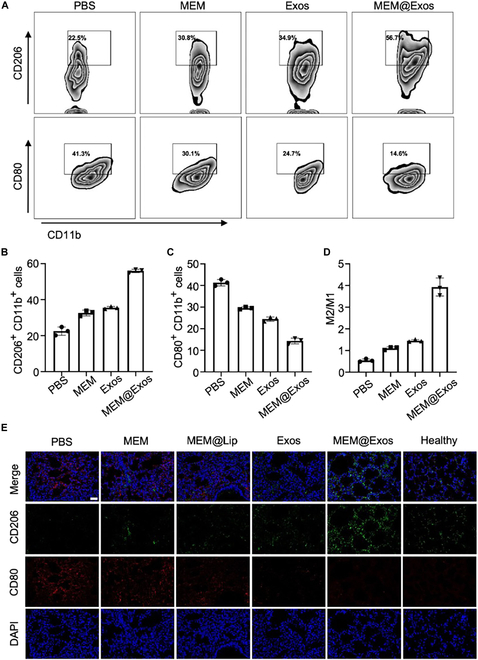
(A) The proportion of CD206/CD11b- and CD80/CD11b-positive cells was analyzed by means of flow cytometry after incubating the *E. coli*-infected M1-types with PBS, MEM, M2-Exos, and MEM@Exos for 24 h, respectively. The percentages of CD206^+^ CD11b^+^ cells (B) and CD80^+^ CD11b^+^ cells (C) after *E. coli*-infected M1-types incubating with PBS, MEM, M2-Exos, and MEM@Exos for 24 h. (D) The proportion of M2-types and M1-types in different groups. (E) The immunofluorescence staining images of CD206 and CD80 using fluorescence microscope in frozen lung tissue of ALI mice after being treated with PBS, MEM, MEM@Lip, M2-Exos, MEM@Exos, and healthy, respectively. Staining: the nucleus (blue), CD206^+^ cells (green), and CD80 (red). Scale bar, 20 μm.

First, as aforementioned, *E. coli*-infected M1-type macrophages were prepared. After incubating the inflammatory macrophages with PBS, MEM, M2-Exos, and MEM@Exos for 24 h, respectively, CD80- and CD206-positive cells were detected by means of flow cytometry. As shown in Fig. 5A, macrophages were dominated by M1 phenotypes (CD80^+^ CD11b^+^) accounting for 41.3%, while M2 phenotypes (CD206^+^ CD11b^+^) only accounted for 22.5%. Compared with PBS group, the proportion of CD80-positive cells in the MEM, M2-Exo, and MEM@Exo groups gradually decreased, while the proportion of CD206-positive cells increased. In the MEM group, the number of M1-types decreased slightly. In the M2-Exo group, the proportion of CD80^+^ CD11b^+^ cells further decreased (24.7%), while CD206^+^ CD11b^+^ cells occupation increased (34.9%), indicating that Exo cloud regulated the polarization of macrophage M1 toward M2. Finally, in the MEM@Exo group, the most pronounced phenotypic shift was observed (CD80^+^/CD206^+^: 14.6%/56.7%), indicating the synergistic regulatory impact of Exos and antibiotics. The bar graph shows that the phenotype of macrophages treated with MEM@Exos was significantly reversed, and the proportion of M2-types was over 4 times higher than that of M1-types (Fig. 5B to D). This Exo-mediated macrophage repolarization was also confirmed by cell morphology changes and cellular factors (Figs. S9 and S10). To visualize the macrophage polarization landscape in the microenvironment of ALI, immunofluorescence staining of CD206 was performed in the ALI lung tissues (Fig. 5E). The results reveal that the expression of CD206 was consistent with the results in vitro. In contrast to the other experimental groups, the Exo-containing group exhibited a notable increase in the number of CD206-stained cells, indicating a prevalence of M2-type macrophages, with the MEM@Exo group displaying the highest proportion (Fig. S11). These findings provide evidence that MEM@Exo nanocomposites have the capacity to reprogram macrophages toward M2-type polarization, fostering the creation of an anti-inflammatory microenvironment. Such an environment is beneficial for mitigating the robust “cytokine storm” associated with M1-type macrophages.

### MEM@Exos ameliorate inflammatory response in ALI mice

**Fig. 6.**
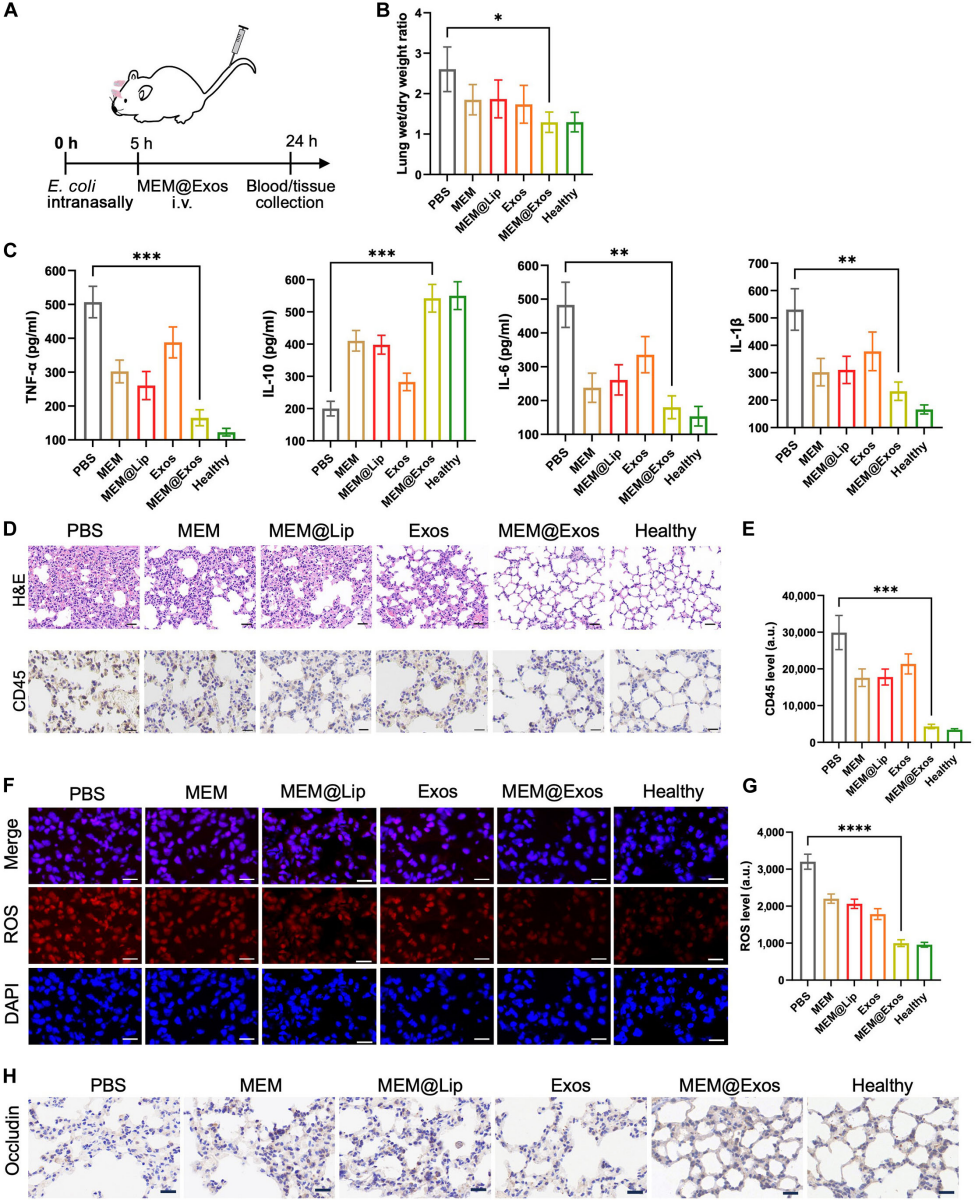
(A) *E. coli* was instilled intranasally into mice for ALI model. Then, ALI mice were intravenously (i.v.) injected with an equimolar amount of PBS, MEM, M2-Exos, and MEM@Exos after 5 h, respectively. Blood and major organs of ALI mice were collected after 24 h. (B) Fresh living lung tissues and lungs dried at 60 °C were weighed, and the ratio of lung dry to wet weight was calculated in PBS, MEM, M2-Exo, and MEM@Exo groups. (C) ALI mice induced by *E. coli* were treated by intravenous administration of PBS, MEM, M2-Exos, and MEM@Exos (20 μg/ml). After 24 h, the serum levels of TNF-α and IL-10 in the 4 groups were detected by ELISA. (D) Images of the H&E staining of lung tissues and leukocyte marker CD45 in lung tissues. Scale bars, 50 μm. (E) The quantitative results of CD45 expression level in PBS, MEM, M2-Exo, and MEM@Exo group. (F) The level of ROS was observed in ALI lung tissues using fluorescence microscope after being treated with PBS, MEM, M2-Exos, and MEM@Exos. Staining: the nucleus (blue) and ROS (red). Scale bars, 50 μm. (G) The quantitative results of ROS expression level in different groups. (H) The expression of the tight junction protein occludin within the lung epithelium of different groups. *P < 0.05, **P < 0.01, ***P < 0.001, and ****P < 0.0001.

To verify the effect of MEM@Exos in repairing ALI in vivo, 40 μl of inoculum of *E. coli* (approximately 4 × 10^9^ colony-forming units) was instilled intranasally into mice for ALI model. As shown in the experimental schedules in Fig. 6A, ALI mice were divided into 4 groups and intravenously injected with an equimolar amount of PBS, MEM, M2-Exos, and MEM@Exos after 5 h, respectively. Blood and main organs of ALI mice were collected for biochemical index evaluation 24 h after successful modeling. The severity of pulmonary edema was assessed by measuring the wet and dry weight of lungs as an indicator of ALI. The results show that for the group treated with MEM@Exos, the ratio of wet/dry weight in lung was the lowest (*P* < 0.05) (Fig. 6B), suggesting that MEM@Exos had alleviated pulmonary edema. In addition, lung tissue was sliced and stained. The staining results of leukocyte common antigen CD45 indicated that the level of CD45 in the MEM@Exo group was the lowest, which meant that the number of infiltrating white blood cells in the injured lung was the lowest (Fig. 6D). The quantitative results of CD45 expression levels show that the reduction was statistically significant (*P* < 0.001) (Fig. 6E).

Further, the peripheral blood of ALI mice was collected, and the expression levels of proinflammatory factor TNF-α and anti-inflammatory factor IL-10 were detected with ELISA kits. As shown in Fig. 6C, the trend of TNF-α secretion was consistent with CD45 level in tissues; that is, with the decrease in inflammatory cell infiltration, it decreased significantly. In contrast, the secretion of IL-10 exhibited a significant upward trend (Fig. 6C). Compared with the PBS control group, this anti-inflammatory trend was statistically different in the M2-Exo group and more significant in the MEM@Exo group (*P* < 0.0001). These findings indicate that M2-Exos possess an intrinsic capacity to regulate inflammatory cells toward anti-inflammatory phenotypes. When MEM was introduced, the combined anti-inflammatory effects of M2-Exos and MEM substantially attenuated the surge of pulmonary inflammatory factors. Previous research has suggested that heightened ROS expression can disrupt the integrity of lung epithelial and endothelial cell barriers, resulting in fluid leakage into the pulmonary parenchyma and extensive infiltration of leukocytes, including neutrophils and macrophages, ultimately contributing to the development of pneumonia [37]. Hence, in the present study, the level of ROS in ALI lung tissues was detected. Obviously, the level of ROS could be inhibited significantly in all groups compared with the PBS control group (Fig. 6F and G). The inhibitory effect was most significant in the MEM@Exo treatment group (*P* < 0.0001). Besides, the tight junction protein occludin expression was significantly preserved in the MEM@Exo-treated group compared to untreated ALI mice (Fig. 6H), indicating enhanced barrier integrity. We further evaluated the therapeutic effects in animal models of intracellular bacterial infection. The results showed that the number of colonies cultured after treatment MEM@Exos was significantly reduced compared with other groups (Fig. S12). Overall, the described in vivo animal experimental results suggest that MEM@Exos could attenuate the inflammatory cytokine storm, relieve alveolar edema, reduce ROS damage, and ultimately treat severe pneumonia without significant side effects to major organs (Fig. S13), which was also confirmed in animal models of MRSA infection (Fig. S14).

## Conclusion

In the present study, an infection-targeting platform to deliver antibiotics for intracellular bactericidal and pneumonia treatment was successfully developed. The developed MEM@Exos not only target the bacterial infection site but also inhibit the production of proinflammatory factors and alleviate inflammatory stress via reprogramming macrophages toward M2-type phenotypes. Notably, MEM@Exos serves as a protective “armor” for the delivery of antibiotics to inflamed macrophages, thereby enhancing antibiotic penetration and efficacy in combating challenging intracellular infections. This bioactive platform holds significant promise as a novel and effective treatment strategy for the management of severe infectious diseases.

Pulmonary drug delivery is a highly effective strategy for treating infectious lung diseases, with macrophages offering significant potential as a target in inflammatory conditions [38–43]. The homotypic recognition of RAW264.7-derived Exos to preferentially target macrophages in an inflammatory microenvironment. This recognition arises from specific surface proteins and membrane markers inherent to macrophage-derived Exos, including integrins, tetraspanins, and other adhesion molecules, which facilitate selective interactions with inflamed macrophages. These molecules enable Exos to recognize and bind to macrophages, particularly those in the activated state present in ALI. In addition to surface markers such as integrins and tetraspanins, the homotypic recognition mechanism is driven by specific molecular interactions between exosomal proteins and macrophage receptors. These interactions facilitate targeted internalization, promoting effective delivery of therapeutic agents into inflammatory macrophages. Previous studies have demonstrated that such Exo–macrophage binding is influenced by both receptor–ligand recognition and the exosomal lipid composition.

The selective delivery of MEM@Exos to macrophages in ALI rather than tissues can be attributed to both biological and physiological factors. In ALI, lung macrophages are highly activated and overexpress receptors and adhesion molecules that strongly bind the exosomal surface markers. This overexpression facilitates preferential accumulation in inflamed lungs compared to other organs. Although Exos naturally have a tropism for the liver, in our study, we observed significant accumulation in the lungs due to the ALI microenvironment. This was confirmed via biodistribution studies (Fig. 3E), which showed increased MEM@Exo uptake in lung tissues compared to other organs, including the liver. In the present study, the pulmonary edema of ALI mice treated with MEM@Exos was significantly improved. The number of infiltrating white blood cells in the injured lung tissues was significantly reduced. The secretion of inflammatory factor TNF-α was significantly decreased, and the anti-inflammatory factor IL-10 was increased. Over time, the initial inflammatory milieu underwent a gradual transformation into an anti-inflammatory microenvironment. To achieve this result, 3 key points were necessary: (a) M2-Exos had good homologous targeting and high immunoaffinity, as demonstrated by the “inflammatory chemotaxis theory” and “immune affinity capture theory” [44,45], which showed good colocalization with the inflammatory sites and supported the notion that Exos constitute communication vehicles between cells [46,47]; (b) M2-Exos had good histocompatibility with its parent macrophages, which facilitated the transport of MEM into the cells. MEM in M2-Exos can effectively kill live *E. coli* after being phagocytosed by macrophages. Moreover, the intracellular bactericidal effect was gradually enhanced with the increased concentration of MEM@Exos and the extension of time. These findings conclusively demonstrate that M2-Exos are capable of effectively transporting exogenous antibiotics into macrophages, aiding in the accomplishment of intracellular sterilization. This ability is attributed to their excellent histocompatibility; (c) M2-Exos had a good immunomodulated function, which could reprogram monocytes toward M2-type polarization. At the same time, the wrapped MEM in M2-Exos could kill *E. coli*, leading to the reduction of endogenous LPS, which helps to weaken the polarization of M1-type. The end result was a lessening of the inflammatory cytokine storm, which is considerably significant for alleviating ALI.

M2-Exos reprogram macrophages via key signaling pathways, including nuclear factor κB, signal transducers and activators of transcription 3, and phosphatidylinositol 3-kinase/Akt, which promote anti-inflammatory polarization. However, we recognize the limitations of this study, including the use of a single bacterial strain (*E. coli*) and the lack of long-term follow-up studies. Potential off-target effects should be addressed in future studies.

For clinical translation, large-scale production of Exos remains a challenge; however, advances in tangential flow filtration and ultracentrifugation are improving yields. Exos exhibit low immunogenicity due to their natural origin, unlike synthetic nanoparticles that may trigger unwanted immune responses. Given the increasing interest in Exo-based therapeutics, regulatory approval pathways must be carefully considered to ensure the safe and effective clinical implementation of MEM@Exos. The potential for combining MEM@Exos with immunotherapy or anti-inflammatory drugs could further enhance their therapeutic efficacy. In addition, the risk of bacterial resistance to antibiotics delivered via Exos can be mitigated by combining therapies or developing new antibiotic formulations.

## Data Availability

All data are available in the main text or the Supplementary Materials.

## References

[1] GBD 2019 LRI Collaborators. Age-sex differences in the global burden of lower respiratory infections and risk factors, 1990-2019: Results from the Global Burden of Disease Study 2019. Lancet Infect Dis. 2022;22(11):1626–1647. 35964613 10.1016/S1473-3099(22)00510-2 PMC9605880

[2] Abed N, Couvreur P. Nanocarriers for antibiotics: A promising solution to treat intracellular bacterial infections. Int J Antimicrob Agents. 2014;43(6):485–496. 24721232 10.1016/j.ijantimicag.2014.02.009

[3] Abdulla A, Dijkstra A, Hunfeld NGM, Endeman H, Bahmany S, Ewoldt TMJ, Muller AE, van Gelder T, Gommers D, Koch BCP. Failure of target attainment of beta-lactam antibiotics in critically ill patients and associated risk factors: A two-center prospective study (EXPAT). Crit Care. 2020;24(1):558. 32933574 10.1186/s13054-020-03272-z PMC7493358

[4] Abdulla A, Ewoldt T, Purmer I-M, Muller AE, Gommers D, Endeman H, Koch B. A narrative review of predictors for β-lactam antibiotic exposure during empirical treatment in critically ill patients. Expert Opin Drug Metab Toxicol. 2021;17(4):359–368. 33463382 10.1080/17425255.2021.1879049

[5] Invernizzi R, Lloyd CM, Molyneaux PL. Respiratory microbiome and epithelial interactions shape immunity in the lungs. Immunology. 2020;160(2):171–182. 32196653 10.1111/imm.13195 PMC7218407

[6] Kalil AC, Metersky ML, Klompas M, Muscedere J, Sweeney DA, Palmer LB, Napolitano LM, O’Grady NP, Bartlett JG, Carratalà J, et al. Executive summary: Management of adults with hospital-acquired and ventilator-associated pneumonia: 2016 Clinical Practice Guidelines by the Infectious Diseases Society of America and the American Thoracic Society. Clin Infect Dis. 2016;63(5):575–582. 27521441 10.1093/cid/ciw504 PMC4981763

[7] Guilhaumou R, Benaboud S, Bennis Y, Dahyot-Fizelier C, Dailly E, Gandia P, Goutelle S, Lefeuvre S, Mongardon N, Roger C, et al. Optimization of the treatment with beta-lactam antibiotics in critically ill patients−Guidelines from the French Society of Pharmacology and Therapeutics (Société Francaise de Pharmacologie et Therapeutique-SFPT) and the French Society of Anaesthesia and Intensive Care Medicine (Société Francaise d’Anesthesie et reanimation-SFAR). Crit Care. 2019;23(1):104. 30925922 10.1186/s13054-019-2378-9 PMC6441232

[8] Beam JE, Rowe SE, Conlon BP. Shooting yourself in the foot: How immune cells induce antibiotic tolerance in microbial pathogens. PLOS Pathog. 2021;17(7): Article e1009660. 34293056 10.1371/journal.ppat.1009660 PMC8297873

[9] Horn J, Stelzner K, Rudel T, Fraunholz M. Inside job: *Staphylococcus aureus* host-pathogen interactions. Int J Med Microbiol. 2018;308(6):607–624. 29217333 10.1016/j.ijmm.2017.11.009

[10] Guo C, Islam R, Zhang S, Fang J. Metabolic reprogramming of macrophages and its involvement in inflammatory diseases. EXCLI J. 2021;20:628–641. 33883988 10.17179/excli2020-3053 PMC8056050

[11] Bhagwat SP, Gigliotti F, Wang J, Wang Z, Notter RH, Murphy PS, Rivera-Escalera F, Malone J, Jordan MB, Elliott MR, et al. Intrinsic programming of alveolar macrophages for protective antifungal innate immunity against *Pneumocystis* infection. Front Immunol. 2018;9:2131. 30283457 10.3389/fimmu.2018.02131 PMC6156154

[12] Wang S, Liu G, Li Y, Pan Y. Metabolic reprogramming induces macrophage polarization in the tumor microenvironment. Front Immunol. 2022;13: Article 840029. 35874739 10.3389/fimmu.2022.840029 PMC9302576

[13] Wang N, Liang H, Zen K. Molecular mechanisms that influence the macrophage m1-m2 polarization balance. Front Immunol. 2014;5:614. 25506346 10.3389/fimmu.2014.00614 PMC4246889

[14] Zubair K, You C, Kwon G, Kang K. Two faces of macrophages: Training and tolerance. Biomedicines. 2021;9(11):1596. 34829825 10.3390/biomedicines9111596 PMC8615871

[15] Arora S, Dev K, Agarwal B, Das P, Syed MA. Macrophages: Their role, activation and polarization in pulmonary diseases. Immunobiology. 2018;223(4-5):383–396. 29146235 10.1016/j.imbio.2017.11.001 PMC7114886

[16] Purushothaman A. Exosomes from cell culture-conditioned medium: Isolation by ultracentrifugation and characterization. Methods Mol Biol. 2019;1952:233–244. 30825179 10.1007/978-1-4939-9133-4_19

[17] Wu Q, Fu S, Xiao H, Du J, Cheng F, Wan S, Zhu H, Li D, Peng F, Ding X, et al. Advances in extracellular vesicle nanotechnology for precision theranostics. Adv Sci. 2023;10(3): Article e2204814. 10.1002/advs.202204814 PMC9875626 36373730

[18] Imran M, Jha S-K, Hasan N, Insaf A, Shrestha J, Shrestha J, Devkota HP, Khan S, Panth N, Warkiani ME, et al. Overcoming multidrug resistance of antibiotics via nanodelivery systems. Pharmaceutics. 2022;14(3):586. 35335962 10.3390/pharmaceutics14030586 PMC8950514

[19] Sahoo S, Kariya T, Ishikawa K. Targeted delivery of therapeutic agents to the heart. Nat Rev Cardiol. 2021;18(6):389–399. 33500578 10.1038/s41569-020-00499-9 PMC8140998

[20] Laffleur B, Batista C-R, Zhang W, Lim J, Yang B, Rossille D, Wu L, Estrella J, Rothschild G, Pefanis E, et al. RNA exosome drives early B cell development via noncoding RNA processing mechanisms. Sci Immunol. 2022;7(72): Article eabn2738. 35658015 10.1126/sciimmunol.abn2738 PMC9357289

[21] Quesenberry P-J, Goldberg LR, Aliotta JM, Dooner MS, Pereira MG, Wen S, Camussi G. Cellular phenotype and extracellular vesicles: Basic and clinical considerations. Stem Cells Dev. 2014;23(13):1429–1436. 24564699 10.1089/scd.2013.0594 PMC4066231

[22] Wiklander O-P, Nordin J-Z, O’Loughlin A, Gustafsson Y, Corso G, Mäger I, Vader P, Lee Y, Sork H, Seow Y, et al. Extracellular vesicle in vivo biodistribution is determined by cell source, route of administration and targeting. J Extracell Vesicles. 2015;4:26316. 25899407 10.3402/jev.v4.26316 PMC4405624

[23] Morad G, Carman CV, Hagedorn EJ, Perlin JR, Zon LI, Mustafaoglu N, Park T-E, Ingber DE, Daisy CC, Moses MA. Tumor-derived extracellular vesicles breach the intact blood-brain barrier via Transcytosis. ACS Nano. 2019;13(12):13853–13865. 31479239 10.1021/acsnano.9b04397 PMC7169949

[24] Hou Y, Liu Y, Liang S, Ding R, Mo S, Yan D, Li D. The novel target: Exosoms derived from M2 macrophage. Int Rev Immunol. 2021;40(3):183–196. 32783545 10.1080/08830185.2020.1800687

[25] Wu G, Zhang J, Zhao Q, Zhuang W, Ding J, Zhang C, Gao H, Pang D-W, Pu K, Xie H-Y. Molecularly engineered macrophage-derived exosomes with inflammation tropism and intrinsic heme biosynthesis for atherosclerosis treatment. Angew Chem Int Ed Engl. 2020;59(10):4068–4074. 31854064 10.1002/anie.201913700

[26] Yang R, Liao Y, Wang L, He P, Hu Y, Yuan D, Wu Z, Sun X. Exosomes derived from M2b macrophages attenuate DSS-induced colitis. Front Immunol. 2019;10:2346. 31749791 10.3389/fimmu.2019.02346 PMC6843072

[27] Joshi AD, Oak SR, Hartigan AJ, Finn WG, Kunkel SL, Duffy KE, Das A, Hogaboam CM. Interleukin-33 contributes to both M1 and M2 chemokine marker expression in human macrophages. BMC Immunol. 2010;11:52. 20958987 10.1186/1471-2172-11-52 PMC2967528

[28] Yuan D, Zhao Y, Banks W-A, Bullock KM, Haney M, Batrakova E, Kabanov AV. Macrophage exosomes as natural nanocarriers for protein delivery to inflamed brain. Biomaterials. 2017;142:1–12. 28715655 10.1016/j.biomaterials.2017.07.011 PMC5603188

[29] Flannagan RS, Cosío G, Grinstein S. Antimicrobial mechanisms of phagocytes and bacterial evasion strategies. Nat Rev Microbiol. 2009;7(5):355–366. 19369951 10.1038/nrmicro2128

[30] Yeh Y-C, Huang T-H, Yang S-C, Chen C-C, Fang J-Y. Nano-based drug delivery or targeting to eradicate bacteria for infection mitigation: A review of recent advances. Front Chem. 2020;8:286. 32391321 10.3389/fchem.2020.00286 PMC7193053

[31] Fraunholz M, Sinha B. Intracellular Staphylococcus aureus: Live-in and let die. Front Cell Infect Microbiol. 2012;2:43. 22919634 10.3389/fcimb.2012.00043 PMC3417557

[32] Virzì GM, Mattiotti M, de Cal M, Ronco C, Zanella M, De Rosa S. Endotoxin in sepsis: Methods for LPS detection and the use of omics techniques. Diagnostics. 2022;13(1):79. 36611371 10.3390/diagnostics13010079 PMC9818564

[33] Geiss C, Salas E, Guevara-Coto J, Regnier-Vigouroux A, Mora-Rodriguez RA. Multistability in macrophage activation pathways and metabolic implications. Cells. 2022;11(3):404. 35159214 10.3390/cells11030404 PMC8834178

[34] Chen X, Liu Y, Gao Y, Shou S, Chai Y. The roles of macrophage polarization in the host immune response to sepsis. Int Immunopharmacol. 2021;96: Article 107791. 34162154 10.1016/j.intimp.2021.107791

[35] Zhou T, Huang Z, Sun X, Zhu X, Zhou L, Li M, Cheng B, Liu X, He C. Microglia polarization with M1/M2 phenotype changes in rd1 mouse model of retinal degeneration. Front Neuroanat. 2017;11:77. 28928639 10.3389/fnana.2017.00077 PMC5591873

[36] Zhang B, Lin F, Dong J, Liu J, Ding Z, Xu J. Peripheral macrophage-derived exosomes promote repair after spinal cord injury by inducing local anti-inflammatory type microglial polarization via increasing autophagy. Int J Biol Sci. 2021;17(5):1339–1352. 33867850 10.7150/ijbs.54302 PMC8040463

[37] Matthay MA, Zemans RL, Zimmerman GA, Arabi YM, Beitler JR, Mercat A, Herridge M, Randolph AG, Calfee CS. Acute respiratory distress syndrome. Nat Rev Dis Primers. 2019;5(1):18. 30872586 10.1038/s41572-019-0069-0 PMC6709677

[38] He S, Gui J, Xiong K, Chen M, Gao H, Fu Y. A roadmap to pulmonary delivery strategies for the treatment of infectious lung diseases. J Nanobiotechnol. 2022;20(1):101. 10.1186/s12951-022-01307-x PMC8892824 35241085

[39] Yu YJ, Yan JH, Chen QW, Qiao JY, Peng SY, Cheng H, Chen M, Zhang XZ. Polymeric nano-system for macrophage reprogramming and intracellular MRSA eradication. J Control Release. 2023;353:591–610. 36503071 10.1016/j.jconrel.2022.12.014

[40] Wang Y, Liu S, Li L, Li L, Zhou X, Wan M, Lou P, Zhao M, Lv K, Yuan Y, et al. Peritoneal M2 macrophage-derived extracellular vesicles as natural multitarget nanotherapeutics to attenuate cytokine storms after severe infections. J Control Release. 2022;349:118–132. 35792186 10.1016/j.jconrel.2022.06.063 PMC9257240

[41] Ma Q, Fan Q, Xu J, Bai J, Han X, Dong Z, Zhou X, Liu Z, Gu Z, Wang C. Calming cytokine storm in pneumonia by targeted delivery of TPCA-1 using platelet-derived extracellular vesicles. Matter. 2020;3(1):287–301. 32835220 10.1016/j.matt.2020.05.017 PMC7242942

[42] Lehar SM, Pillow T, Xu M, Staben L, Kajihara KK, Vandlen R, DePalatis L, Raab H, Hazenbos WL, Morisaki JH, et al. Novel antibody–antibiotic conjugate eliminates intracellular *S. aureus*. Nature. 2015;527(7578):323–328. 26536114 10.1038/nature16057

[43] Wan S, Wang K, Huang P, Guo X, Liu W, Li Y, Zhang J, Li Z, Song J, Yang W, et al. Mechanoelectronic stimulation of autologous extracellular vesicle biosynthesis implant for gut microbiota modulation. Nat Commun. 2024;15(1):3343. 38637580 10.1038/s41467-024-47710-w PMC11026491

[44] Ax E, Jevnikar Z, Cvjetkovic A, Malmhall C, Olsson H, Radinger M, Lasser C. T2 and T17 cytokines alter the cargo and function of airway epithelium-derived extracellular vesicles. Respir Res. 2020;21(1):155. 32560723 10.1186/s12931-020-01402-3 PMC7304225

[45] Doyle L-M, Wang M-Z. Overview of extracellular vesicles, their origin, composition, purpose, and methods for exosome isolation and analysis. Cells. 2019;8(7):727. 31311206 10.3390/cells8070727 PMC6678302

[46] Zhu S, Huang H, Liu D, Wen S, Shen L, Lin Q. Augmented cellular uptake and homologous targeting of exosome-based drug loaded IOL for posterior capsular opacification prevention and biosafety improvement. Bioact Mater. 2022;15:469–481. 35386342 10.1016/j.bioactmat.2022.02.019 PMC8958386

[47] Shen Z, Kuang S, Zhang Y, Yang M, Qin W, Shi X, Lin Z. Chitosan hydrogel incorporated with dental pulp stem cell-derived exosomes alleviates periodontitis in mice via a macrophage-dependent mechanism. Bioact Mater. 2020;5(4):1113–1126. 32743122 10.1016/j.bioactmat.2020.07.002 PMC7371600

