# Supplementary material for: Molecular Targeting of Intracellular Bacteria by Homotypic Recognizing Nanovesicles for Infected Pneumonia Treatment
Source: Biomater Res. 2025 Apr 2;29:0172. doi: 10.34133/bmr.0172 (PMC11964281; doi:10.34133/bmr.0172)
Supplement: Supplementary 1 — Figs. S1 to S14 [file bmr.0172.f1.docx]

Supporting Information

Molecular targeting of intracellular bacteria by homotypic recognizing nanovesicles for infected pneumonia treatment

*Xu Wang^1^, Hao Zhou^2^, Dan Li^3^, Zhe Zhao^4^, Ke Peng^5^, Xiang Xu^6^, Jia-Jia Wang^7^,* *Yang Wang^1^,* *Jun Wang^8^, Jing-Jing Zhang^9^, Shuang-Shuang Wan^9^, Mai-Qing Shi^1^, Jun Chen^1*^,* *Xian-Guang Ding^9*^, and Fu-Hai Ji**^*5^*


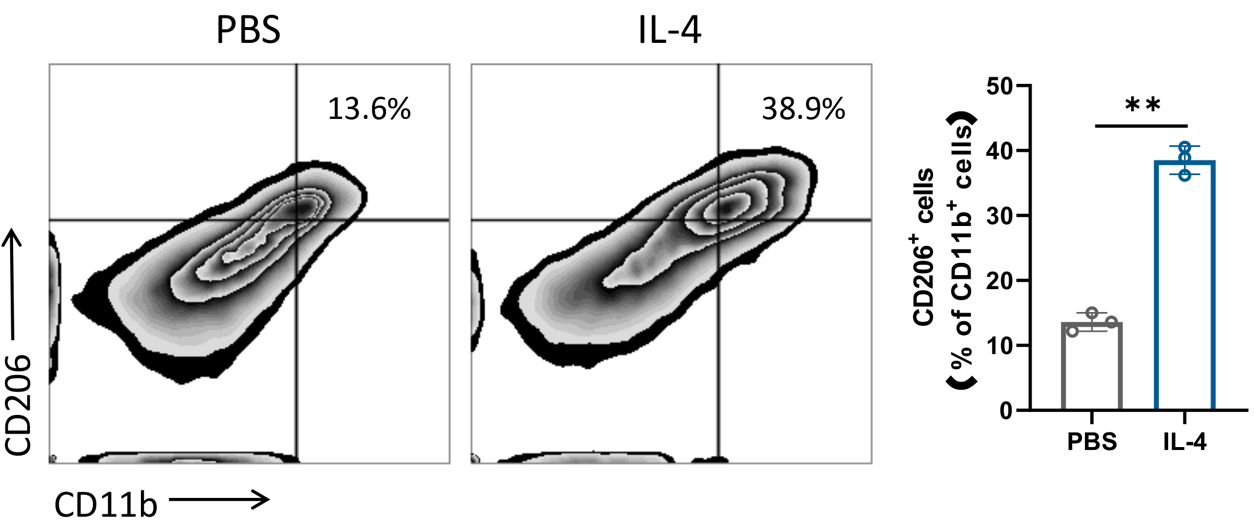


**Figure S1.** The expression of typical M2-type biomarker CD206 was detected by flow cytometry after RAW 264.7 macrophages stimulated by IL-4 or PBS for 12 h. The percentage of CD206+CD11b+ cells in different groups (p<0.01).


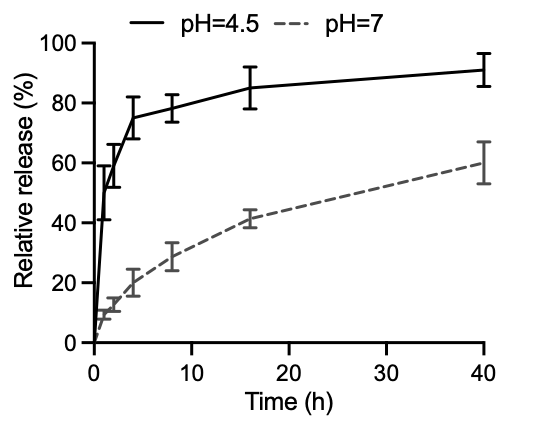


**Figure S2.** The release profile of the percentage of MEM from Exos under different pH conditions. The pH value of 4.5 is used for the mimic endolysosomal condition, and the pH value of 7 is used for the mimic physiological condition.


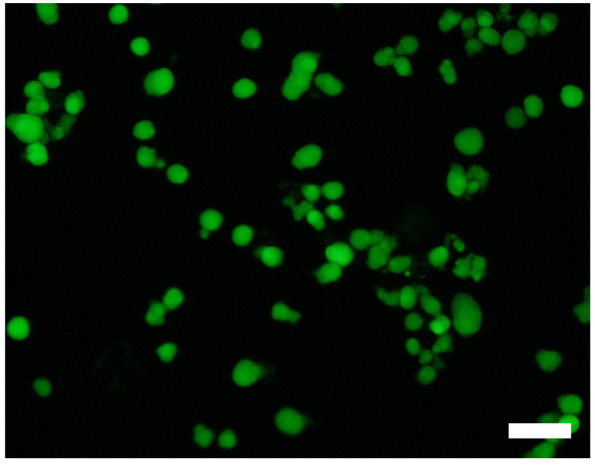


**Figure S3.** The activity of HUVECs stained by AM/PI was observed after being treated with MEM@Exos using fluorescence microscope. Red: dead cells. Green: live cells. Scale bar, 50 μm.


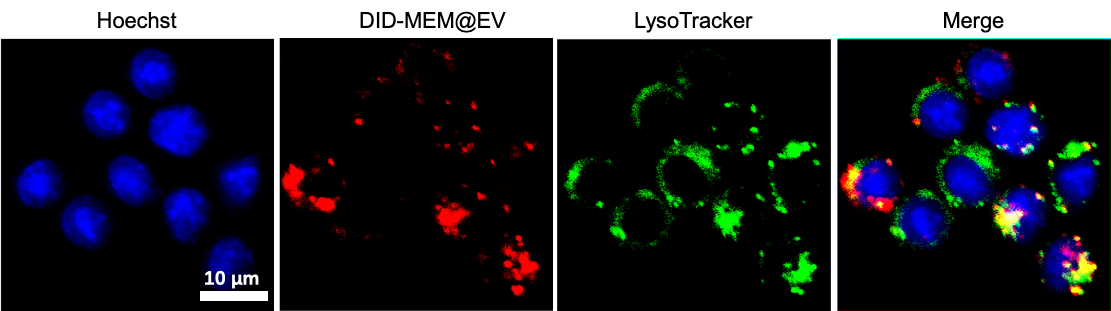


Figure S4. Fluorescence-labeled MEM@EV for tracking their internalization within macrophages.

Figure S5. The pharmacokinetics MEM@Exos.


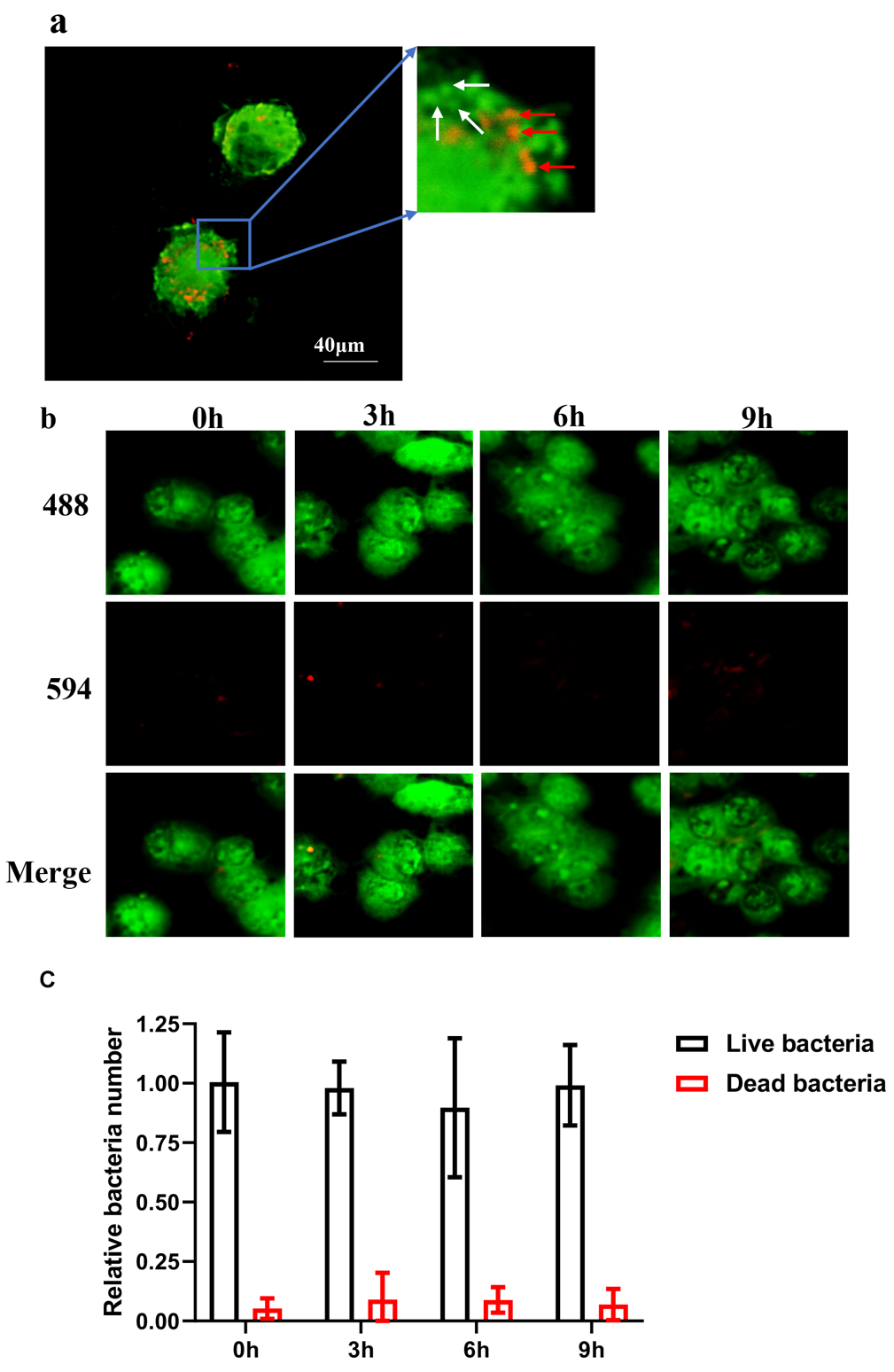


**Figure S6.** a. RAW264.7 cells infected with E. coli were observed by laser cofocus microscopy after staining by the LIVE/DEAD BacLight kit. Dead bacteria (red, red arrows) and live bacteria (green, white arrows). b. E. coli-infected macrophages were co-cultured with free MEM for 0, 3, 6 and 9 hours, and then stained with a LIVE/DEAD BacLight kit. Red: dead bacteria. Green: live bacteria. c. The relative number of live and dead bacteria at different times (0, 3, 6 and 9 h).


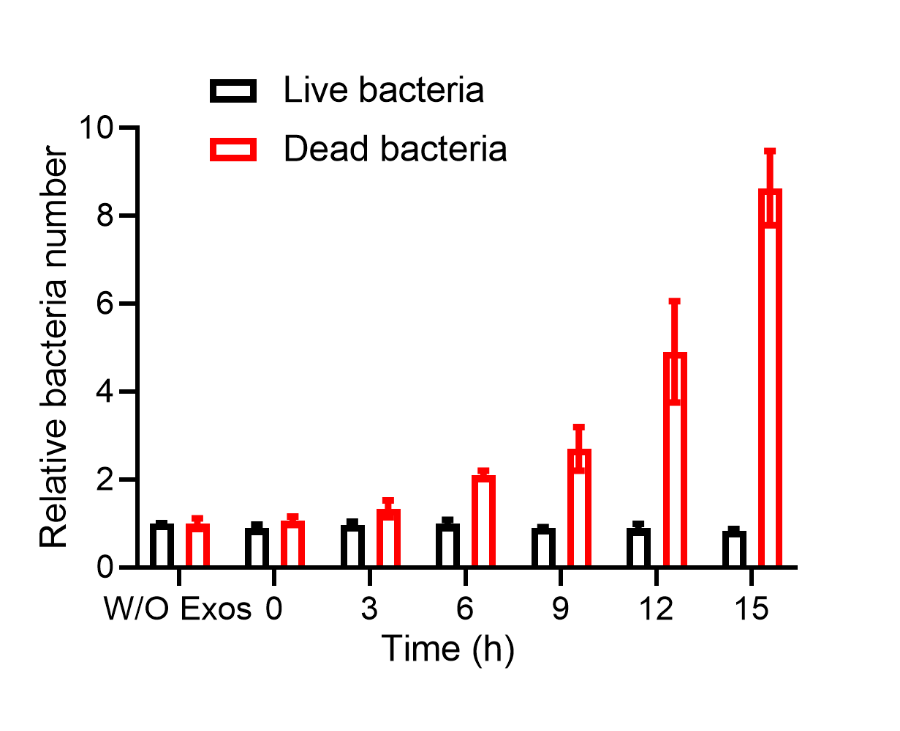


**Figure S7.** The infected macrophages with E. coli were co-cultured with MEM@Exos (20 µg/mL equiv. MEM) for 0, 3, 6, 9, 12 and 15 hours. The relative number of live and dead bacteria was shown.


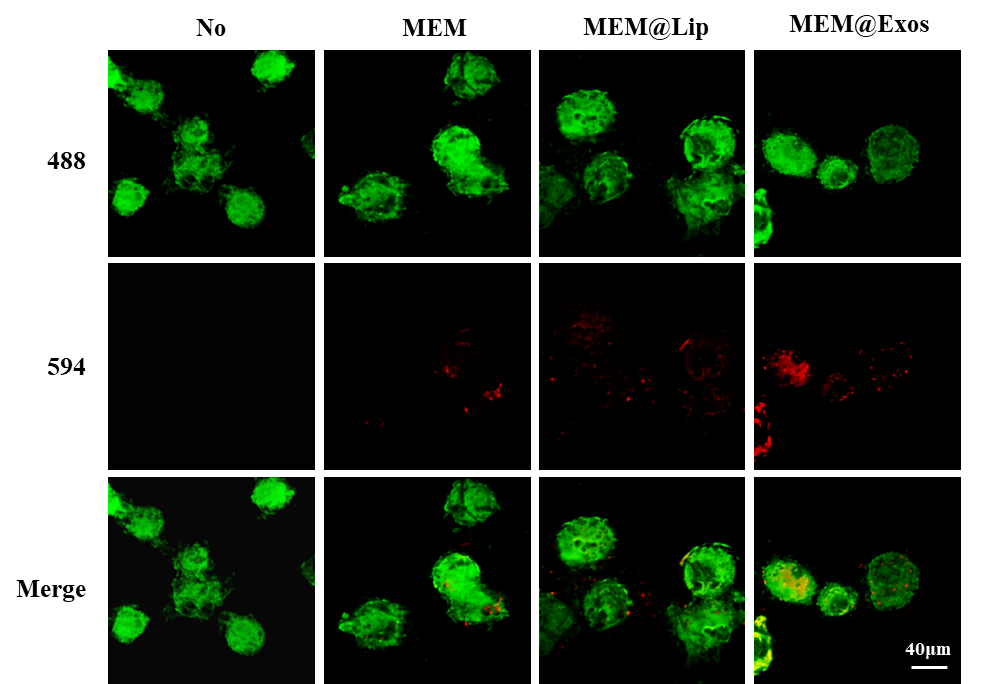


**Figure S8.** The infected macrophages by S. aureus (MRSA) were co-cultured with MEM@Exos and subsequently stained using the LIVE/DEAD BacLight kit. Dead bacteria are labeled in red, and live bacteria are labeled in green. Scale bar: 40 μm.

**Figure S9.** After being treated with
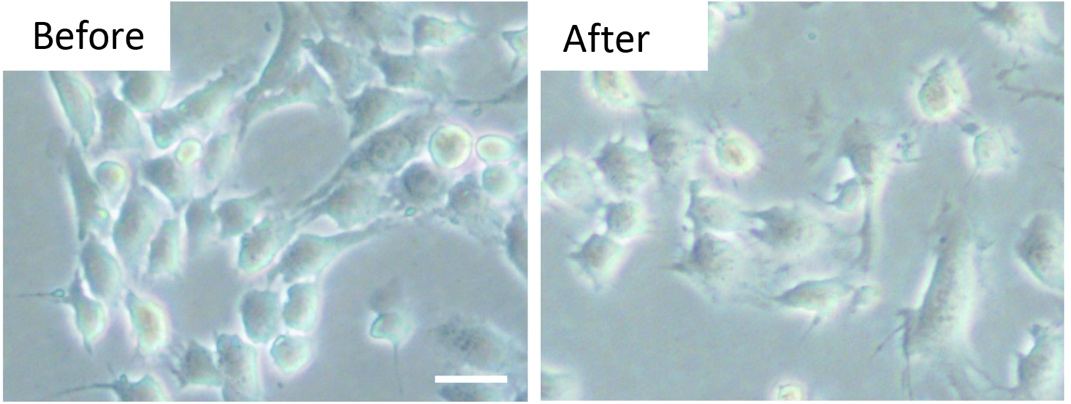
MEM@Exos, the morphology change of E. coli-infected M1-types was clearly observed. Scale bar, 20 μm.


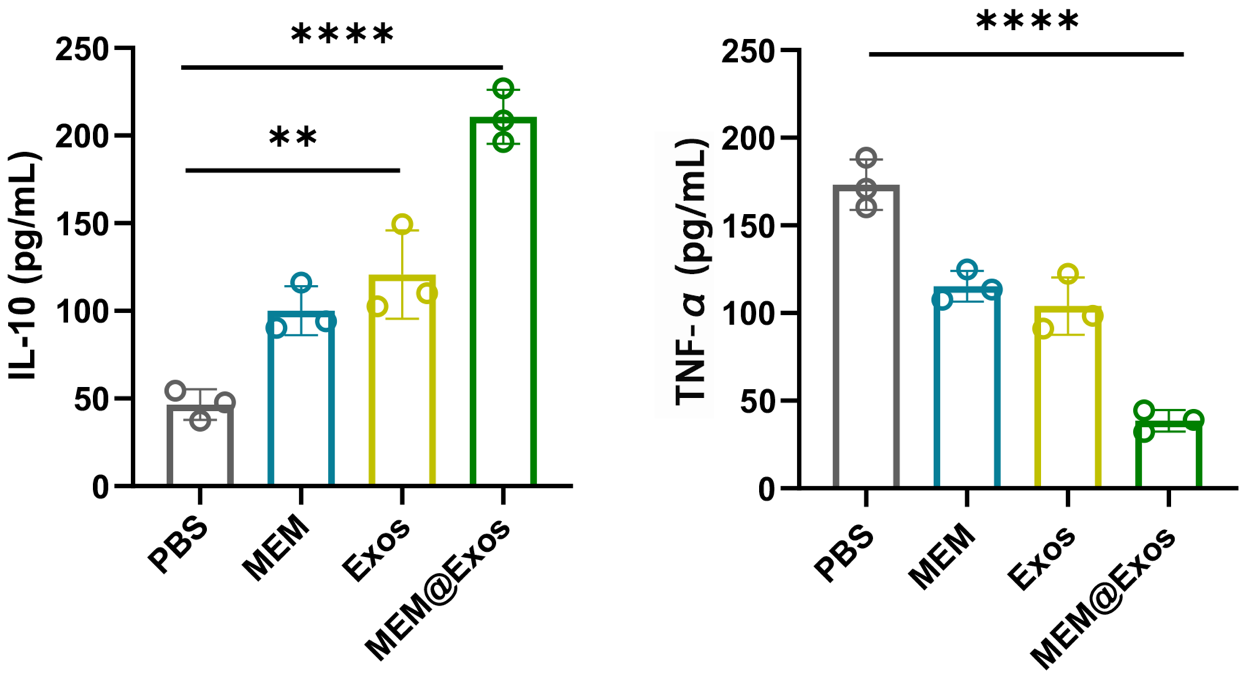


**Figure S10.** The level of IL-10 and TNF-α was detected after incubating E. coli-infected M1-types with PBS, MEM, M2-Exos and MEM@Exos for 24 h by ELISA (IL-10: p<0.0001 and TNF-α: p<0.0001).


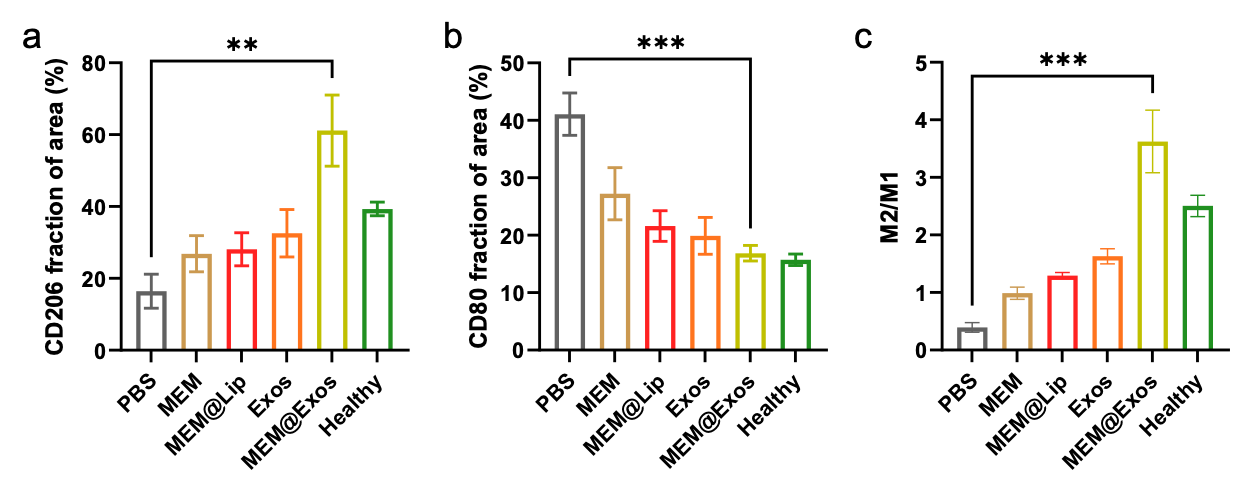


**Figure S11.** Quantification of the number of CD206+ and CD80+ cells in immunostaining.


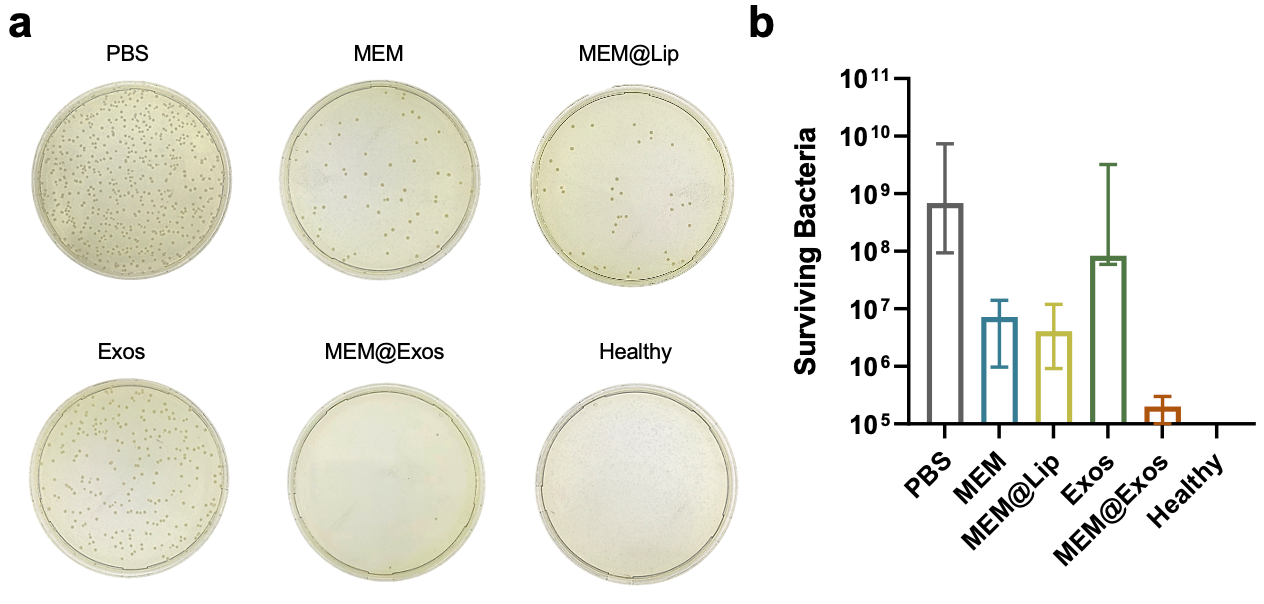


**Figure S12.** The therapeutic effects in animal models of intracellular bacterial infection.


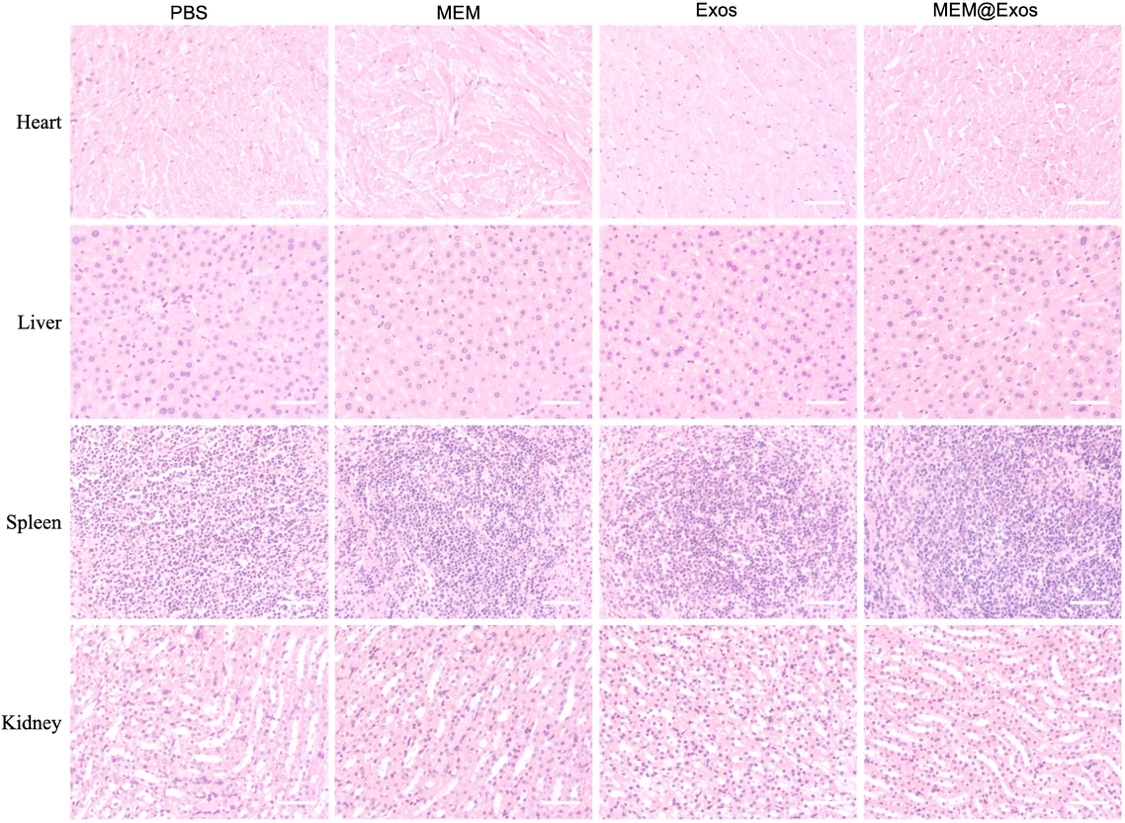


**Figure S13.** Major organs were sliced for HE staining, including the heart, liver, spleen, and kidney, after PBS, MEM, M2-Exos, and MEM@Exos treated E. coli-induced ALI mice for 24 h.


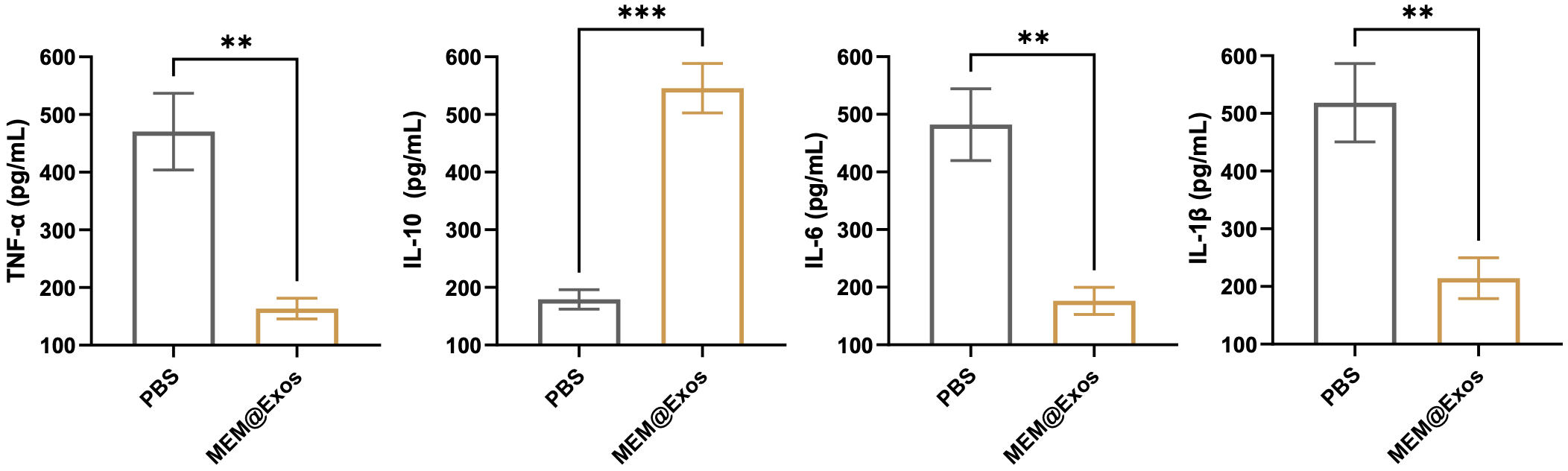


**Figure S14**. The inflammatory effects in animal models of MRSA infection.
